# Sampling High-Dimensional Conformational Free Energy Landscapes of Active Pharmaceutical Ingredients

**DOI:** 10.1021/acs.jctc.5c01247

**Published:** 2025-12-04

**Authors:** Alexandre Ferreira, Rui Guo, Ivan Marziano, Matteo Salvalaglio

**Affiliations:** † Thomas Young Centre and Department of Chemical Engineering, 4919 University College London, London Wc1e 7je, United Kingdom; ‡ 97470 Pfizer Worldwide Research & Development, Sandwich Ct13 9nd, United Kingdom

## Abstract

We present a gridless framework for computing high-dimensional conformational free energy surfaces (FES) of flexible molecules using enhanced sampling trajectories. By combining concurrent well-tempered metadynamics with Density Peaks Advanced (DPA) clustering, our approach bypasses the dimensionality limitations of conventional grid-based FES reconstruction. Free energies are assigned on a per-configuration basis via local density estimation and Zwanzig reweighting, allowing for a direct, resolution-independent mapping of the conformational ensemble. Conformers are identified as density peaks in torsional angle space, and convergence is assessed via systematic consistency metrics. We validate this approach by reproducing the paradigmatic FES of alanine dipeptide and extend it to explore molecules with 4-, 7-, and 11-dimensional torsional angle spaces. As a key application, we investigate the solvent-dependent conformational preferences of bicalutamide in vacuum, chloroform, and DMSO. The predicted global minima reflect the known solvent-induced conformational shift between open and closed forms, in agreement with NMR and crystallographic data. These results demonstrate that our workflow provides a scalable route to high-dimensional conformational free energy landscapes, with direct relevance for polymorphism, solvation, and drug design.

## Graphical abstract

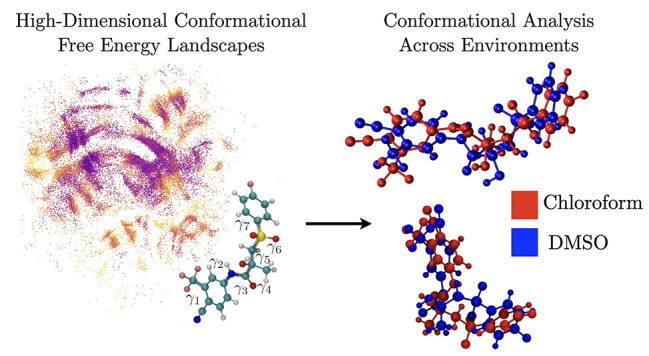

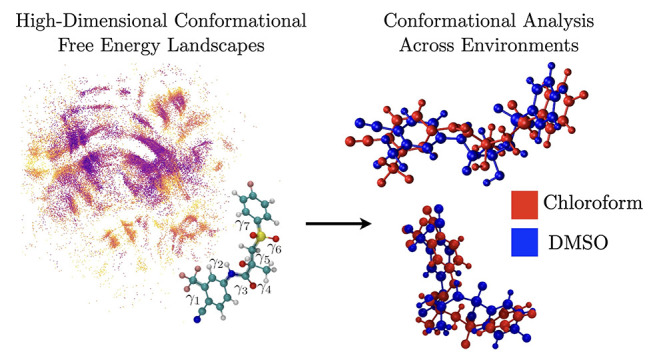

## Introduction

The vast majority of active pharmaceutical ingredients (APIs) are highly flexible molecules, capable of changing shape readily in biological environments, as well as displaying conformational polymorphism in the solid state.^1,2^ This polymorphism is particularly important to the drug formulation and manufacturing process, as the pharmacological properties of APIs can depend very strongly on the crystal’s polymorphism.^3^ As such, quantitatively characterizing the conformational landscape of APIs is of great interest. For instance, it is known that the environment in which a molecule is found impacts its conformational landscape^4–6^ however, a systematic approach for mapping, understanding, and quantifying the effects of the environment on the conformational landscape of APIs is still lacking.^7^ This gap is partly due to the inherent high-dimensionality of these spaces,^8^ which renders their exploration and rationalization challenging, especially when it becomes necessary to explicitly account for the impact of the environment in which an API molecule is found. Here, we present a workflow that combines enhanced sampling molecular dynamics techniques with density-based clustering to simultaneously explore the conformational free energy landscape of flexible API, explicitly accounting for the effects of the environment, and obtain an estimate of the relative free energy of the conformers discovered. These two aspects differentiate our proposed approach from methods based on locally minimizing the potential energy of isolated molecules, where entropic contributions are typically included *a-posteriori* and usually do not include the configurational contribution associated with an explicitly represented environment.^9–12^

Before delving into the details of the method, we introduce the definition of conformational space adopted in this work. A molecule’s conformation can be defined using the values of the molecule’s freely rotatable dihedral angles^1,6^ (henceforth referred to as torsions). The conformational space is, therefore, always bounded and periodic in all dimensions. Veber’s rules,^13^ a set of heuristics initially designed to predict whether a molecular structure would possess pharmacological properties, can be used to identify the relevant torsions in any given molecular structure. Characterizing conformers through the values of a set of torsions is not without precedent, and several approaches are based on this definition. For example, Torsiflex^14^ is a software package that aims to explore a single molecule’s potential energy surface utilizing a semirandom exploration of conformational spaces defined by torsions.

Molecular dynamics (MD) simulations can be used to sample the conformational space and map out the conformational FES of a given molecule. Figure 1 shows different extents of MD sampling of the same collective variable (CV) space. Sampling the probability distribution with MD offers several key advantages; MD sampling is inherently physics-based and enables simulating a molecule in various environments and conditions. The physics-based nature of the sampling means the distribution sampled in conformational space by the MD simulation can be converted into a FES^15^ through the relationship:

1 $$F\left(S\right)=-k_{B}T\;\ln p\left(S\right)$$

where **S** represents the dihedral angle space **S** = [γ_1_, γ_2_, ···, γ_*D*_], where γ_*i*_ is one of the *D* torsional angles of a given molecule. *p*(**S**) is the probability distribution in the conformational space, *F*(**S**) is therefore the FES in conformational space **S**, *k*_B_ is Boltzmann’s constant, and *T* is the temperature.

**Figure 1:**
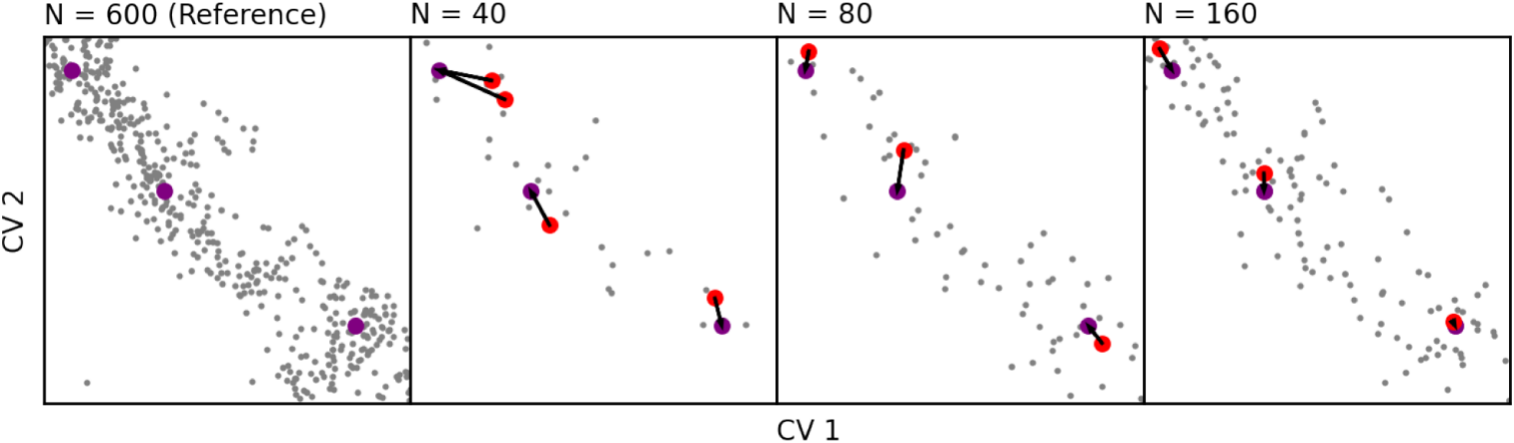
Sketch illustrating the pairing procedure used by the consistency metrics to compare cluster-sets generated from data sets of different sizes. The configurations shown are drawn from the simulation of alanine dipeptide, but the principle illustrated is general. The cluster centers obtained from the largest amount of data form the reference set, shown in purple. Cluster centers generated from smaller amounts of data (shown in red) are paired to the nearest cluster center in the reference set, allowing comparison of distances and energy differences between cluster centers. It is expected that as the data set size grows, the positions and energies of the cluster centers will converge, as seen in Figure 2g,h.

To compute *F*(**S**), it is necessary to obtain an estimate of *p*(**S**), where all energetically relevant regions are ergodically sampled. Achieving an ergodic sampling of relevant configurations is a well-known problem that several enhanced sampling techniques have addressed.

For the ergodic sampling of a well-defined configuration space, metadynamics, which involves depositing penalty biases dynamically as the simulation proceeds to promote sampling,^16^ would be a typical approach. However, for the exhaustive sampling of a conformation space, this approach is limited by the computational feasibility of storing the bias values on a grid of the same dimensionality as the conformation space, which is the same issue faced with the conventional method of FES construction. For this reason, in practical applications, conventional metadynamics biases constructed in dimensionalities higher than three are very rare. Alternatives for high-dimensional CV spaces, such as bias-exchange metadynamics,^17^ have been developed. Still, these generally depend on the running of multiple replica simulations overseen by an exchange scheme. To widely sample conformational space with a single simulation, concurrent^18^ well-tempered^19^ metadynamics (WTMetaD) is used here.

Figure 2a shows a FES for alanine dipeptide computed from a nonconcurrent WTMetaD simulation using what will be referred to as the conventional method.^20^ The WTMetaD biases are deposited in the 2D conformational space, defined by two torsions, ϕ and ψ (as illustrated in Figure 2d), ensuring that the entire space is fully sampled over the course of the simulation. The space is split into a 100 × 100 bins histogram. The distribution of MD configurations throughout the histogram follows the system’s equilibrium probability distribution as distorted by the metadynamics bias. The total bias deposited in each bin is known, allowing this distortion of the probability distribution to be reweighted. The resulting FES has a resolution equal to the fineness of the grid, in this case (2π)/100 rad. This methodology is robust and widely adopted, but scales poorly to higher-dimensional FESes. Both the bias deposition during the simulation and the estimate of the probability distribution require the construction of a grid with the same dimensionality as the conformation space. If the same resolution is desired, increasing the number of torsions incurs an exponential cost on computational resources, rapidly becoming unfeasible. In the scientific literature, this issue is addressed by employing dimensionality reduction methods such as Sketch-map.^21,22^ In these methods, the conformational probability density is obtained by histogramming sampled configurations in a low-dimensional space of unphysical coordinates. While this approach can be effective for relatively small systems,^22^ its ability to resolve degeneracies and identify *conformers* when the conformational spaces are defined by a large number of torsional degrees of freedom is not straightforward.

**Figure 2:**
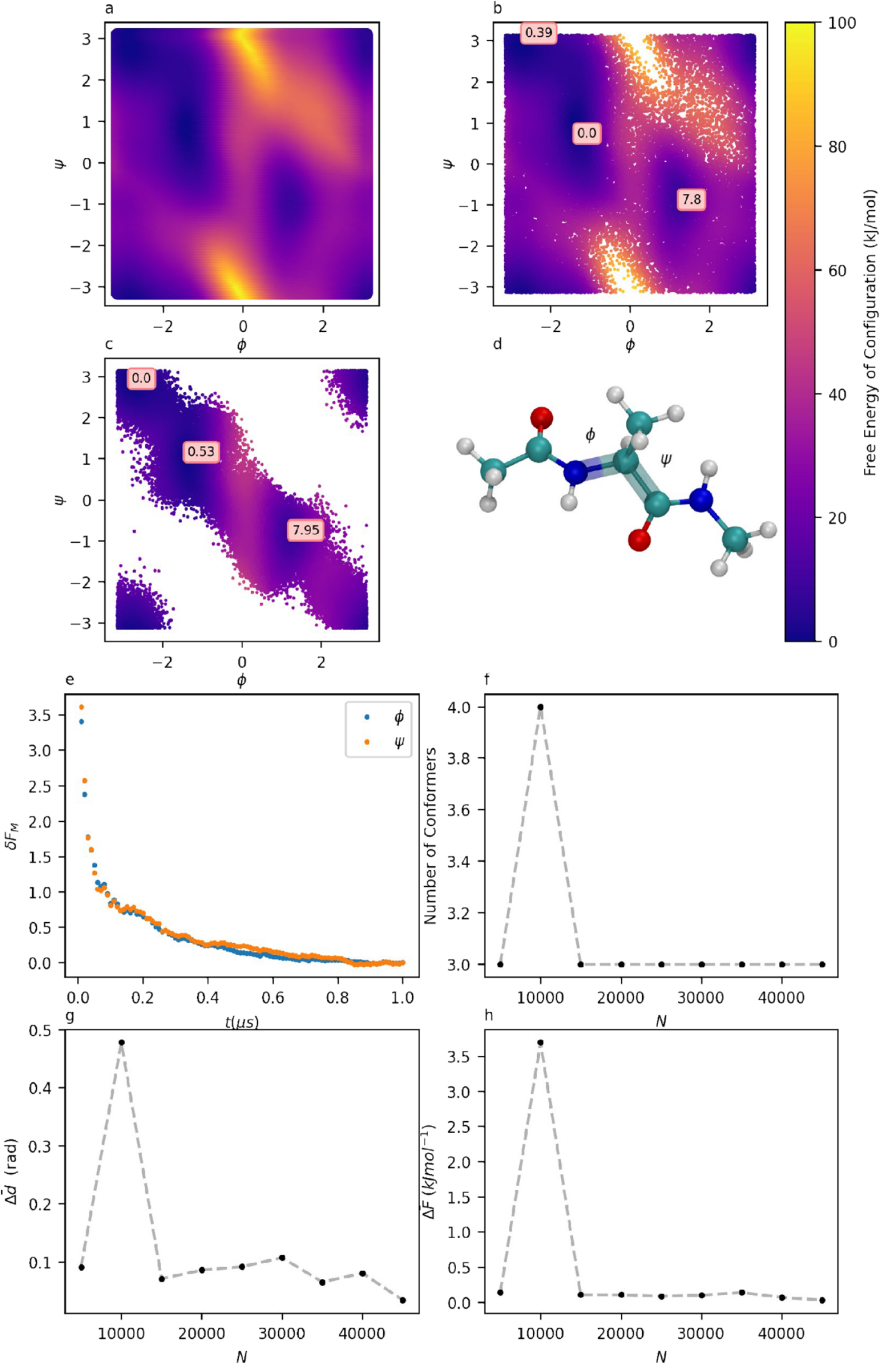
a: A conformational free energy surface for alanine dipeptide, obtained conventionally, through constructing a reweighted probability distribution on a histogram. b: The same free energy surface, constructed from a probability distribution derived from the local densities of individual configurations sampled from the simulation. The positions and free energies of local minima, as identified by DPA, are overlaid. c: The same free energy surface, also constructed from local densities, sampling a simulation using 2 × 1D WTmetaD biases instead of a conventional 2D bias. The positions and free energies of local minima, as identified by DPA, are overlaid. d: Alanine dipeptide, with the two relevant torsions ϕ and ψ highlighted. For a, b, and c, the energies of the FESes are indicated by a colormap in units of kJ mol^–1^. e: Evolution of δ*F*(*t*) on the marginal free energies of each torsion in alanine dipeptide. f: Number of clusters identified by clustering on data sets of size *N*. g: Evolution of Δ*d̅* with *N* for alanine dipeptide. h: Evolution of Δ*F̅* with *N* for alanine dipeptide.

### Biasing and Estimating Probabilities in High Dimensions

#### Gridless Probabilities with Density Peaks Advanced

Density-based clustering techniques form a family of unsupervised machine learning algorithms that group data points within spatial data sets into clusters based on the distance between data points within the data space. Algorithms in this family include DBSCAN^23^ and Fast Search and Find of Density Peaks (FSFDP).^24^ There is precedent for the use of FSFDP in molecular conformation spaces, Marinova et al. used it to study the conformation space of Sildenafil.^6^ Here, Density Peaks Advanced (DPA),^25^ a successor to FSFDP, is used.

DPA, developed by d’Errico et al., splits a set of data points distributed in space into clusters by grouping points within density peaks, a term referring to regions of high data density (N.B. In this work, the term “density” refers to the density of data points in **S**, unless otherwise noted). It does this partly by calculating the local density of every region centered on every single point in the data set. This calculation is a function of the Euclidean distances between the point and its nearest neighbors. Here, the process is applied to a sample of *N* configurations in conformation space sampled by the MD simulation. These local density calculations are extremely powerful in this context for two key reasons: first, each additional dimension in **S** adds a single term to the Euclidean distance calculation, so the cost with increasing dimensionality scales linearly, and second these local densities map a distribution in much the same way as the previously described histogram, so the same reweighing and inversion procedure may be applied to calculate the free energy. These free energies, unlike those in the above histogram, are not associated with a defined region of conformation space. Rather, they are associated with a specific configuration sampled by the simulation. Thus, this *per point* FES has no fixed spatial resolution; data is rich in regions that have been heavily sampled and sparse in regions that have not been frequented. This is advantageous as it means that while data in the local minima remains highly dense due to the frequent sampling, little cost is incurred in considering data from the rarely visited, largely irrelevant high-energy regions. This contrasts with the grid-based approach, where these high-energy regions are modeled in as high a resolution as the more relevant local minima. The results of applying this approach to alanine dipeptide can be seen in Figure 2b.

### Biasing in High-Dimensional Spaces with Concurrent Metadynamics

To avoid the exponentially increasing costs of depositing WTMetaD biases in a high-dimensional conformational space, concurrent metadynamics is used in its place to promote sampling. This entails simultaneously depositing a single one-dimensional bias for each torsion in the molecule, thus encouraging exploration of the rotation of that torsion.^16^ The cost of this approach scales linearly with dimensionality (shown in Figure S1a), as one additional monodimensional grid is needed for each additional torsion considered. The cost savings of this approach come with a trade-off; conventional metadynamics promotes the exploration of the entire conformational phase space and guarantees that previously visited configurations will be penalized accordingly. Concurrent metadynamics does not explicitly bias the combinations of any torsion values. Instead, it promotes the escaping from local free energy wells by driving the rotation of individual torsions. This can be seen in the use of this technique on alanine dipeptide in Figure 2c.

This work will demonstrate that DPA analysis of data sets generated using concurrent WTmetaD can be used to model the probability distribution of a flexible molecule’s conformational state and create a “per-point” FES, where the data points themselves are configurations sampled by the simulation. This approach will be shown to recreate the well-studied 2D FES of alanine dipeptide before being demonstrated on 4- and 11-dimensional conformational free energy landscapes in vacuum and a 7-dimensional conformational free energy landscape in different solution environments.

## Theoretical Background and Methods

### Clustering and FES Construction

To discuss the approach developed in this work in detail, it is helpful to begin by introducing how DPA allows the estimation of a FES from unbiased MD-generated data. Density Peaks Advanced (DPA) is run on a subset of configurations sampled from an MD trajectory. The size of this subset is the limiting factor in the cost of this approach, as each point has a local density determined by the distance to its neighbors, so the process depends on constructing a complete distance matrix between all configuration pairs. We, therefore, expect the cost of this approach to scale approximately with the square of the data set size (see Figure S1b). More advanced implementations that compute distances of *k* neighbors can achieve scaling closer to *N* log *N*, with N being the size of the data set.^26^ Because the thermodynamics of the system directs the MD trajectory’s sampling, the local density of each configuration is proportional to the relative probability of encountering this configuration. It thus can be directly inverted to the free energy of the configuration using eq 1.

DPA estimates the probability associated with the ensemble of configurations projected in a given point *i* of the configuration space **S**, using the PA*k* density estimator,^27^ based on the Euclidean distances between point *i* and its *k* nearest neighbors. An underpinning assumption of this method is that the density is constant in the neighborhood of the point *i*. Hence, the parameter *k* is selected to be as large as possible to maximize the data used in calculating the local density while still representing a hypervolume of constant density. Each neighbor *l* can be said to occupy the volume *v*_*l*_ of the hyperspherical shell enclosed between hyperspheres of radii *r*_*l*_ and *r*_*l*–1_. The sum of these volumes up to neighbor *k* is equal to the volume *V*_*k*_ of a hypersphere with radius *r*_*k*_. DPA leverages the fact that for a region of constant density, the volumes will be drawn from an exponential distribution^28^ with a rate of this density ρ and that thus the log-likelihood function of ρ given a set of *k* neighbors is provided by

2 $$L_{i,k}\left(\rho \right)=k\;\log\left(\rho \right)-\rho V_{k}$$

The PA*k* estimator selects an appropriate *k* value using two models with distinct assumptions. Model one, *M*1, assumes that the densities of point *i* and its *j* = *k* + 1 nearest neighbor are independent, while model two, *M*2, assumes these densities are identical. Their log-likelihood functions are

3 $$L_{M1}=k\;\log\left(\frac{k^{2}}{V_{k}V_{j}}\right)-2k$$

4 $$L_{M2}=2k\;\log\left(\frac{2k}{V_{k}+V_{j}}\right)-2k$$

The two models are compared with a likelihood ratio test,^29^

5 $$D_{k}=-2\left(L_{M2}-L_{M1}\right)$$

which increases as the two models differ. If *D*_*k*_ grows over a threshold *D*_thr_ (*D*_thr_ = 23.928 according to ref 25) then the densities of *i* and *j* cannot be considered constant. As such, PA*k* selects an appropriate *k* value by iteratively calculating *D*_*k*_ for increasing values of *k* until the threshold is passed.

### Per Point Free Energies with DPA and Biased Simulations

In practice, we generate conformational data sets using WTmetaD simulations. Using PA*k* on configurations sampled from a WTmetaD simulation produces densities that reflect a probability distribution perturbed by the applied biases. These densities can be reweighed using the Zwanzig approach^15^ as

6 $$\rho_{i}^{*}=\rho_{i}\times e^{\beta \left(\sum_{t=1}^{D}V_{i}^{t}\right)}$$

where ρ* is the reweighed density, β is equal to 1/*k*_B_ *T*, $V_{i}^{t}$ is the bias in torsion *t*, $\overset{D}{\underset{t=1}{\sum }}V_{i}^{t}$ represents the sum of the concurrent biases acting on the *D* torsions, for configuration *i*. Practically, we evaluate ρ, the biased density, from configurations generated in a quasi-static bias regime, as the bulk of the bias is deposited during the early stages of the simulation, and the bulk of sampled configurations are visited when bias deposition is negligible. We therefore apply the *final bias* approximation to obtain a time-independent value of $V_{i}^{t}$ acting on configuration *i*.^30–32^ To mitigate the noise introduced by exponential reweighting,^15^ the density of each point is then averaged over hyperspherical domains of radius 0.1 rad. This step generates a new smoothed set of densities ${\mathrm{\rho ̅}}_{i}^{*}$ , at the cost of a slight controllable loss in spatial resolution. The free energy *F*_*i*_, associated with configuration *i* (thus termed *per point*), is computed as $F_{i}=-k_{B}T\ln{\mathrm{\rho ̅}}_{i}^{*}$ .

### Conformer Classification

In the classification step, DPA identifies peaks in the density as cluster centers, i.e., distinct conformers. This operation is equivalent to identifying local minima in the *D*-dimensional free energy surface. For this step, we use the set of reweighted, smoothed densities ${\mathrm{\rho ̅}}_{i}^{*}$ .

Moreover, to avoid every fluctuation in density from being identified as a distinct peak, the DPA classifier is set to merge clusters separated by a saddle point between the free energy basins (a conformational transition state) with free energy less than 1 *kT* higher than one of the cluster centers it connects. Once cluster centers have been determined, all remaining configurations are assigned membership to the same cluster as their nearest neighbor of higher density.^25^ With all configurations classified, clusters with a population smaller than 1% of the total sample are discarded to avoid spurious clusters identified from anomalously isolated configurations.

The ultimate product of this process is a set of cluster center configurations representing the local minima of the *D*-dimensional conformational FES, called a cluster set. Each cluster’s lowest free energy configuration, i.e., the cluster center, provides *the most* representative configuration of a given conformer.

### Consistency Analysis

The potential high dimensionality of **S** makes the visualization of per-point free energies difficult. A series of checks on the ergodicity of the sampling and consistency of the DPA classification, therefore, provides confidence in the results.

First, the convergence of the *D* monodimensional marginal free energy surfaces of each torsion is monitored to assess the ergodicity of the sampling. For a simulation of length τ, convergence of the marginals is assessed by monitoring, on *D* histogams of *n*_hist_ points, the quantity:

7 $$\delta F_{M}\left(t\right)=n_{\mathrm{hist}}^{-1}\overset{n_{\mathrm{hist}}}{\underset{i=1}{\sum }}\left|F_{i}^{\tau }-F_{i}^{t}\right|$$

Where simulation time *t* runs from [0,τ], *F*(*t*) is a monodimensional FES obtained with data gathered up to time *t*, *F*(τ) is the same quantity computed with all the data available. This quantity represents the average free energy difference per histogram bin in any of the *D* monodimensional free energy surfaces.

Figure 2e displays an example of δ*F*_*M*_(*t*) computed for ϕ and ψ torsional angles of alanine dipeptide during concurrent metadynamics. The flattening of these differences as the fraction of utilized trajectory increases indicates that the simulation has been run for a sufficiently long time, allowing these torsions to be ergodically sampled.

This check is computationally inexpensive and provides a first qualitative assessment of the quality of the configurational exploration obtained with concurrent WTmetaD. If the marginal FES associated with a torsion is still evolving rapidly at time τ, i.e., when the simulation ends, the sampling has not yet reached the ergodic limit with respect to the configurations discovered.

However, the convergence of 1D marginal FESes tells us little about exploring the conformational space in its full dimensionality. This is important as even substantial amounts of data may be distributed extremely sparsely in high dimensions.

As such, to build confidence in our results, we evaluate the statistical significance of the conformer classification as a function of the data set size.

For this purpose, a consistency check has been devised, which offers a similarity score between two cluster sets generated from different configurations. A cluster set generated from a data set of size *N*, *C* ^*N*^ can be compared with a reference cluster set *C* ^*ref*^, which is generated with the largest number of configurations feasible. Each cluster center $C_{i}^{N}$ is matched with the nearest center in the reference set $C_{i}^{ref}$ , according to the Euclidean distances in **S** between members of the two cluster sets. This matching process is demonstrated in Figure 1. Differences in free energy Δ*F*_*i*_ and position Δ*d*_*i*_ are determined and averaged across all matched pairs as Δ*F̅^N^* and Δ*d̅^N^*. This comparison to *C* ^*ref*^ can be repeated for cluster-sets generated from data sets of increasing *N*, and evolution of Δ*F̅^N^* and Δ*d̅^N^* with growing *N* can thus be assessed. Once data sets are large enough, the positions and relative free energies of minima would be expected to be independent of data set size. The results of this analysis on the case of alanine dipeptide are shown in Figure 2f,g,h.

### Simulation Setup

Unless otherwise specified, all simulations carried out for this work consisted of a single molecule in vacuum, simulated with a 2 fs time step. GAFF^33^ force field parameters were used, and GROMACS^34^ was the MD engine used. WTMetaD was carried out using the Plumed^35^ plugin for GROMACS. The simulations were carried out in the NVT ensemble, at a temperature of 300 K, maintained using the velocity-rescaling thermostat developed by Bussi et al.^36^

For the determination of WTMetaD parameters, a short 10 ns unbiased simulation was run. The marginal FES in each torsion was computed. A Gaussian Mixture Model was fitted to the resulting FES, and the smallest width parameter of the GMM, corresponding to the narrowest local minimum in the marginal FES, was considered as the minimum reference width for the marginal under consideration. The width of the Gaussian terms used to update the metadynamics bias was set to a quarter of the minimum reference width. Following the completion of the WTMetaD simulation, configurations from the simulation were paired with the total deposited bias at the corresponding position in conformation space, which aligned with the final bias approximation.

## Results and Discussion

### Method Validation: Alanine Dipeptide

The workflow outlined in this work was first tested on the Ramachandran plot^37^ of alanine dipeptide. This system was chosen for several reasons: the Ramachandran plot of alanine dipeptide is a commonly used model system in the field of molecular dynamics and enhanced sampling techniques, making it one of the best-studied conformational FESes available. Additionally, its low dimensionality allows for both the visualization of the FES and access to more conventional methods of exploring this conformational space. The principal results of this are shown in Figure 2. The structure of alanine dipeptide, with ϕ and ψ, indicated, is shown in Figure 2d. The conventional FES of alanine dipeptide, obtained through histogramming and reweighing of a trajectory generated through a WTMetaD MD simulation,^20^ is shown in Figure 2a. A per-point FES, generated from the same simulation but with free energies calculated using the local densities of the sampled configurations, as discussed in the Methods section, is shown in Figure 2b. From a visual comparison, it clearly appears that the two FESes are in agreement, demonstrating that the reweighted-DPA density estimate leads to results virtually indistinguishable from standard histogramming-based approaches. Figure 2c shows a per-point FES generated using a trajectory from a concurrent metadynamics simulation where ϕ and ψ are biased independently. Besides demonstrating the consistency of the free energies obtained by concurrent biasing, the comparison between Figure 2b and Figure 2c illustrates the trade-offs entailed by using concurrent metadynamics. As detailed in the Methods section, concurrent metadynamics promotes the sampling of metastable states without guaranteeing an exhaustive sampling of the joint configurational probability density. Nevertheless, all relevant free energy minima are adequately sampled, and their positions and free energies agree with those obtained by standard, two-dimensional metadynamics (Figure 2).

The results of the consistency analysis techniques outlined in the Methods section on the per-point FES outlined in Figure 2c are shown on Figure 2e–h. Figure 2e shows the evolution of δ*F*(*t*) for ϕ and ψ. The flattening of the curves shows that the sampling of the two torsions is indeed ergodic over the time scale of the simulation.

Figure 2h shows mean conformer free energy difference Δ*F̅^N^* (defined in the Methods section) obtained from clustering data sets of increasing *N* and a reference data set at *N* = 50,000 configurations. Figure 2g shows a similar plot displaying the mean separation of the cluster centers, Δ*d̅*. Figure 2f shows the number of minima identified by DPA for each reduced-size data set. The plot shows that all reduced data sets agreed that there were 3 conformers, with the exception of the 10,000 configuration data set. In all other data sets, there is very good agreement on the position and free energies of the local minima, with energy differences well within 1 kJ mol^–1^ and mean separations hovering around 0.1 rad. It is surprising how little data is required to generate reasonable results in this 2-dimensional case. Even in Figure 1, where very small data sets are used to illustrate the cluster-set matching procedure, the agreement in minima positions is apparent.

### Applications to Higher-Dimensional Free Energy Surfaces

Having demonstrated the workflow developed here on the two-dimensional case of alanine dipeptide , higher-dimensional cases are now explored, where visualization of the entire conformation space is not possible, and conventional grid-based methods become unfeasible. Sulfadiazine, with a 4-dimensional conformational space, and Candidate XXXII, from the CSP Blind Test,^8,38^ with an 11-dimensional conformational space, serve as a test for the ability of the workflow to handle conformational complexity. Sketch-map^21^ is used in these cases to project a 2D representation of the high-dimensional per-point FES for human interpretation.

#### Sulfadiazine

Sulfadiazine is an antibiotic molecule with a 4-dimensional conformational space; its clinical relevance and intermediate complexity make it an ideal next step for the method outlined here. A 4-dimensional space is too high to allow a FES to be fully visualized while still being low enough that reasonable data density can be obtained (50,000 data points in a periodic 4D space results in an average density of roughly 32 configurations per *rad* ^4^). The inset in Figure 3a shows the 4 torsions considered in sulfadiazine. Using the same approach outlined above for alanine dipeptide, sulfadiazine’s conformational FES was studied by analyzing the configurations sampled within a 1 μs single-molecule WTmetaD simulation. The resulting per-point FES cannot be fully visualized without dimensionality reduction, so the relative free energies and coordinates of each minimum are presented in Table 1. Figure 3a shows a 2D projection of the 4D per-point FES created using SketchMap. This representation preserves the short-distance connectivity between data points, allowing for the visualization of distinct free energy basins and the transition states between them, although the two axes of the new 2D projection are not physically meaningful in themselves.^21^ It should be emphasized that the estimation of densities and the determination of the number and coordinates of the free energy minima are determined in the full 4-dimensional conformation space and that the projection in Figure 3a serves only to assist in the visualization of the relationships between different conformers. It is possible to combine the 4-dimensional information presented in Table 1 with the 2-dimensional intuition provided by Figure 3a. For example, the FES in Figure 3a appears to be bisected by a diagonal channel, and indeed, by inspecting the torsion values of the conformer pairs 17 and 2, 10 and 6, 21 and 15, and 7 and 8, it can be determined that these conformers pairs are equivalent, and differ from each other in a symmetric rotation of π radians of γ_3_. This example illustrates how these 2D projections can be interpreted and demonstrates how symmetry elements in the molecule’s conformational space can be preserved in the 2D projection.

**Figure 3:**
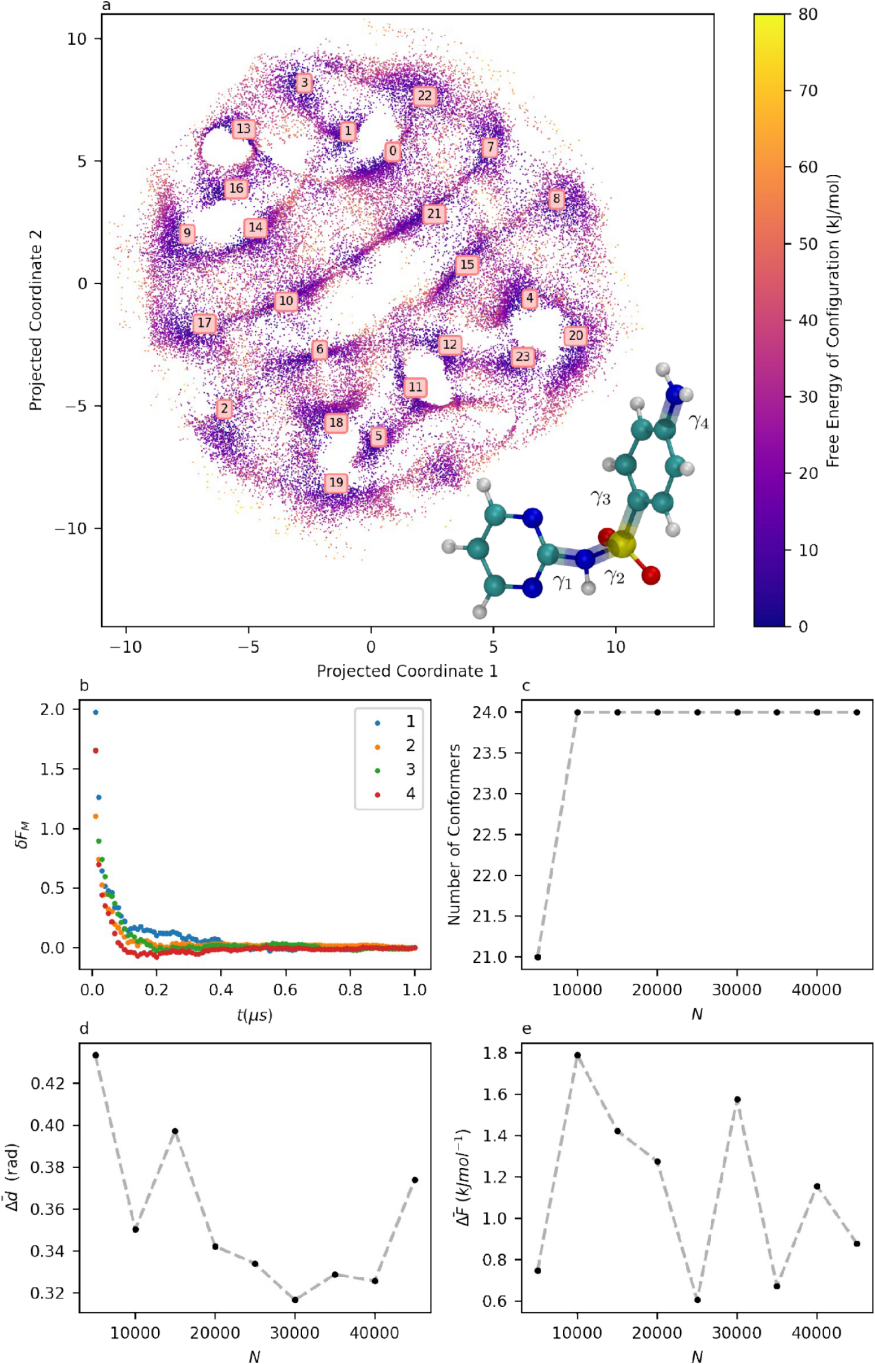
a: 2D Sketch-Map projection of sulfadiazine’s 4D conformation surface, with molecular structure of SDZ inset. Distances between configurations are preserved over small separations, but the axes themselves have no physical meaning. b: Evolution of δ*F*(*t*) on the marginal free energies of each torsion in sulfadiazine. c: Number of clusters identified by clustering on data sets of size *N*. d: Evolution of Δ*d̅* with *N* for sulfadiazine. e: Evolution of Δ*F̅* with *N* for sulfadiazine.

**Table 1:** Labels, Free Energies, and CV-Space Coordinates of Sulfadiazine’s 24 Conformers^a^

Conformer	Free energy [kJ/mol]	γ_1_	γ_2_	γ_3_	γ_4_
0	0.72	–1.71	–2.1	–1.51	2.95
1	1.28	–1.7	0.13	–1.52	–2.98
2	0.75	1.55	–1.83	1.54	0.21
3	1.44	–1.62	–0.13	–1.47	–0.05
4	0.08	–1.81	–1.94	1.62	–2.93
5	1.05	1.64	0.09	1.58	2.86
6	0.0	1.92	1.75	1.42	–0.28
7	1.03	–1.43	1.93	–1.31	0.07
8	1.66	–1.49	2.08	1.26	0.23
9	0.67	1.3	–1.87	–1.6	–3.02
10	0.63	1.68	2.05	–1.66	0.15
11	1.56	1.62	–0.11	1.68	0.21
12	1.81	–1.55	0.01	1.64	0.17
13	0.21	1.62	–0.16	–1.65	–0.15
14	0.93	1.85	1.89	–1.53	3.13
15	1.02	–1.76	–1.95	1.88	0.31
16	1.2	1.62	0.06	–1.7	–3.08
17	1.4	1.4	–1.96	–1.77	0.15
18	1.58	1.74	1.88	1.63	–3.07
19	0.21	1.55	–2.04	1.83	2.93
20	0.8	–1.55	2.0	1.54	–2.81
21	0.47	–1.82	–1.83	–1.59	0.2
22	1.76	–1.46	2.0	–1.77	2.92
23	1.03	–1.58	0.01	1.69	2.9

a The labeling convention is consistent with that of Figure 3.

The results of the consistency metrics for sulfadiazine are shown in Figure 3b–e. In comparing these results to those in Figure 2e–h, it is possible to evaluate the impact of doubling the dimensionality of the conformation space on the accuracy and data efficiency of the classification process. The plots of δ*F*(*t*) for the four torsions show that the four marginals in Figure 3b converge rapidly, providing evidence of ergodicity. Figure 3c shows that, except for the 5000-point data set, repeated analyses achieve a consistent number of 24 conformers. Figure 3d,e shows the evolution of Δ*F̅* and Δ*d̅* respectively, as *N* increases to a reference value of 50,000. Here, the differences between alanine dipeptide and sulfadiazine become apparent. The mean free energy deviation jumps from being nearly negligible to a range between 0.5 and 2 kJ mol^–1^, and positional deviation increases from approximately 0.1 rad to between 0.3 and 0.4 rad. Sulfadiazine’s energy deviation is still within 1 *k*_B_T, and the positional deviations still correspond to very small changes in the molecular structure. However, the abrupt change following an increase in dimensionality highlights the importance of carrying out consistency checks when working with highly unintuitive results that are difficult to inspect visually. Due to the number of equivalent conformers related to one another by symmetric transformations in sulfadiazine, it is possible to compare the free energies of equivalent conformers as an assessment of the reproducibility of the free energy calculation. This is not recommended as a standard practice, as the presence of symmetrically related conformers is system-dependent and not guaranteed. However, in this case, comparing the differences between equivalent conformers reveals deviations of the same order as the mean free energy deviations calculated in the smaller data sets (Figure 3e).

#### Target XXXII of the Seventh CCDC Blind Test

The highest-dimensional conformational FES explored here is that of Molecule XXXII, a target from the seventh CSP Blind Test.^8,38^ As a highly flexible drug-like molecule with a conformation space defined by 11 torsions (shown in the inset in Figure 4a), it is chosen here to test the limits of our method. To facilitate comparison with results collected for alanine dipeptide and sulfadiazine, the results presented here were generated using consistent simulation and analysis parameters. Using 50,000 data points in this high-dimensional space results in an average data density of approximately 8 × 10^–5^ configurations per rad^11^. Despite the extremely low data density, which is inherently linked to the complexity of the conformational space, we show that meaningful results are achievable. Figure 4a shows the projected 11-dimensional per-point FES, with cluster centers corresponding to 11-dimensional coordinates presented in Table 2. When comparing this FES to sulfadiazine’s in Figure 3a, the features of XXXII can be seen reflected in its own FES. The relative lack of symmetrical torsions results in a less symmetrical FES, and the higher-dimensional FES is much sparser, illustrating that the computational savings arise from a more efficient, rather than more exhaustive, sampling of conformational space.

**Figure 4:**
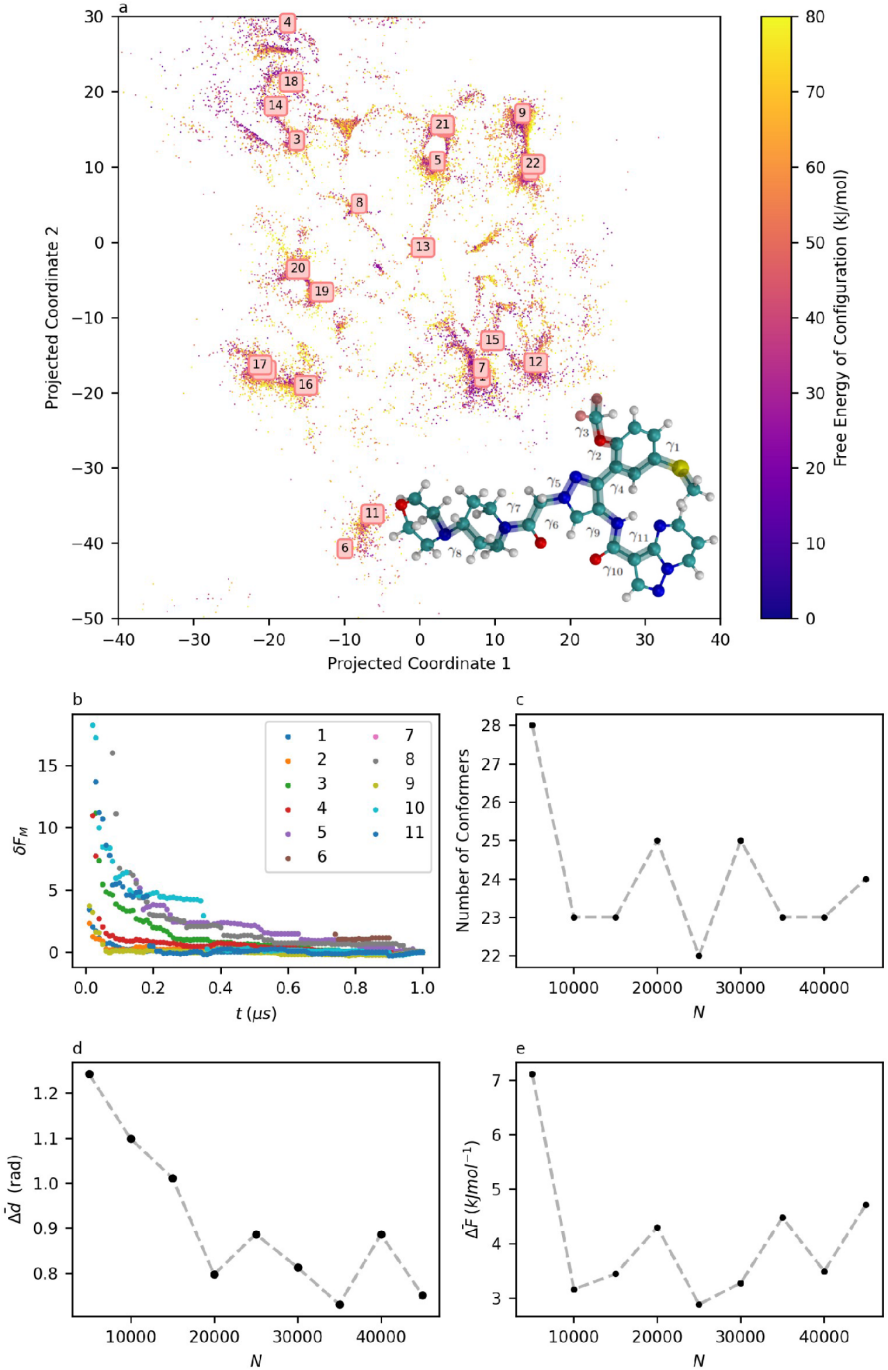
a: 2D Sketch-Map projection of XXXII’s 11D conformation surface, with molecular structure of XXXII inset. Distances between configurations are preserved over small separations but the axes themselves have no physical meaning. b: Evolution of δ*F*(*t*) on the marginal free energies of each torsion in XXXII. c: Number of clusters identified by clustering on data sets of size *N*. d: Evolution of Δ*d̅* with *N* for XXXII. e: Evolution of Δ*F̅* with *N* for XXXII.

**Table 2:** Labels, Free Energies, and CV-Space Coordinates of XXXII’s 23 Conformers^a^

Conformer	Free energy [kJ/mol]	γ_1_	γ_2_	γ_3_	γ_4_	γ_5_	γ_6_	γ_7_	γ_8_	γ_9_	γ_10_	γ_11_
0	6.09	1.12	–1.27	0.89	0.34	–2.31	1.13	0.42	1.29	2.96	3.13	0.15
1	0.0	3.06	1.84	–1.52	–2.75	–2.24	1.29	0.2	1.12	2.95	–3.13	0.04
2	8.45	1.03	1.98	1.07	0.37	–2.24	1.12	0.85	–1.19	–3.05	3.09	0.12
3	6.2	1.25	1.24	1.05	–2.93	2.43	–1.11	2.65	–1.12	3.11	3.11	–0.08
4	3.4	1.21	–1.05	1.72	2.95	2.23	–1.21	2.28	1.1	–3.09	2.93	–0.1
5	8.69	1.17	1.71	0.85	0.58	–2.06	1.21	–0.22	1.19	–3.06	–2.94	0.2
6	15.67	3.04	–0.8	–1.39	–0.41	1.06	0.93	0.43	1.42	3.07	3.13	–0.34
7	2.17	–3.06	1.14	–1.6	–2.85	–2.0	1.39	–0.18	1.0	3.1	–2.94	0.09
8	16.12	1.07	–0.62	1.61	2.67	0.91	1.01	0.26	0.94	–3.04	3.02	–0.3
9	7.62	1.03	–1.75	0.93	0.56	–2.35	1.25	0.69	–1.06	2.97	–3.08	0.18
10	12.45	–3.09	–0.61	–1.43	–0.3	2.28	–1.13	2.41	1.11	2.99	3.09	–0.18
11	21.03	2.81	1.0	–1.16	–0.36	1.04	1.0	0.51	1.14	2.92	–3.02	–0.31
12	1.43	–3.1	1.39	–1.58	–2.64	–2.34	1.17	0.64	–0.89	–3.03	3.12	0.07
13	6.92	–2.99	1.63	–1.6	–2.7	–1.02	–0.97	2.73	–0.93	2.83	–2.92	0.04
14	5.96	1.17	–0.86	1.51	2.86	1.96	–0.94	2.64	–1.04	3.12	–3.05	–0.09
15	5.72	3.04	0.71	–1.49	–2.51	–2.39	1.24	0.53	3.03	3.03	–2.98	–0.09
16	10.98	–3.01	2.07	–0.91	–0.57	2.26	–1.36	2.17	1.23	3.13	3.09	–0.16
17	8.91	–3.1	–1.4	–0.9	–0.42	2.12	–1.16	2.32	1.26	3.03	–2.94	–0.12
18	6.88	1.18	–1.62	1.61	2.66	2.1	–1.19	2.85	–0.95	–3.12	2.92	0.03
19	9.53	3.02	1.55	–1.25	–0.56	2.09	–1.2	2.69	–0.91	2.98	3.11	–0.09
20	6.89	3.07	–2.12	–1.31	–0.62	2.07	–1.17	2.86	–1.06	3.06	3.11	–0.21
21	7.35	1.18	–2.3	1.2	0.55	–2.11	0.89	0.41	1.37	2.86	–2.81	0.09
22	6.13	1.16	1.23	0.85	0.27	–2.15	1.19	0.59	–0.89	3.14	–2.93	0.16

a The labeling convention is consistent with that of Figure 3.

The consistency metrics in Figure 4b–e are, however, less reliable than those obtained for sulfadiazine. Figure 4b shows well-converged marginal free energies, but Figure 4c shows that the number of conformers identified is less consistent than in lower-dimensional cases. The number of metastable states identified as distinct conformers hovers between 22 and 25 for data sets sized 10000 and upward. Along with a fluctuating number of conformers, larger deviations in free energies and positions are now observed, with Δ*F̅* between cluster sets now varying by up to 5 kJ mol^–1^, and Δ*d̅* drifting by as much one full radian, even at large data set sizes. Despite this drop in the quality of the results, we believe it is still remarkable that a reasonably intuitive understanding of such a high-dimensional conformational FES can be derived from a limited amount of data in a computationally accessible way, even if its value in this instance is chiefly qualitative. To further explore the consistency of the FES in Figures 4, and S2–S10 contain the FES projection for each of the smaller data sets used in the consistency analysis, allowing the evolution of this per-point FES to be observed. Inspection of this evolution in the FES appears to reveal that the majority of fluctuations in Δ*F̅* and Δ*d̅* observed arise in higher-energy conformers, with the low-energy regions converging at lower *N* values. Although we do not rigorously prove this here, it is a reasonable expectation, as lower energy regions have a high data density, resulting in more accurate free energy estimates based on a greater amount of data.

### Exploring the Impact of Solvent on the Conformational Landscapes of Bicalutamide

DPA operates on a set of molecular configurations defined purely by the values of the subject molecule’s torsions. As such, the cost of the analysis is independent of the length, complexity, and level of theory of the simulations from which the configurational data set is produced. Generating a high-dimensional conformational free energy landscape for a molecule simulated in a solvent environment is, therefore, accessible. In this section, a study of the molecule bicalutamide is presented, examining the impact of two different solvent environments on the conformational free-energy landscape.

Bicalutamide is an antiandrogen compound used for the treatment of prostate cancer. It is highly flexible, with a conformational space described by the 7 dihedral angles shown inset in Figure 7a. This flexibility results in two conformational polymorphs being observed in the solid state: form I, as shown in Figure 9c, and form II, as shown in Figure 9d. Form I demonstrates an open conformation, while form II adopts a more compact, closed conformation. Form I is the more thermodynamically stable form, and is the form which typically arises upon recrystallization from most solvents.^39^ Form II can be obtained from a melt of form I.^40^ Despite form I’s tendency to recrystallize out of most solvents, Sobornova et al. discovered that solvent choice had a significant impact on bicalutamide’s conformational distribution in solution.^39^ Using NOE (nuclear Overhauser effect) spectroscopy, they demonstrated that polar solvents promote an open conformation, while nonpolar solvents promote a closed conformation. Here, the conformational free energy landscapes of bicalutamide, simulated in vacuum, chloroform, and DMSO environments, are explored using the gridless method developed in this study. As before, data sets of 50,000 configurations were used to construct the per-point FESes shown here.

Figure 5 shows the evolution of δ*F*_*m*_(*t*) for each of the 7 torsions of bicalutamide in vacuum, dichloromethane, and DMSO. This figure demonstrates the convergence of each of these one-dimensional marginal free energies. The results of the higher dimensional consistency checks are shown in Figure 6. Figure 6a shows that a consistent number of conformers was not reached in any of the environments simulated. This inconsistency in conformer number is characteristic of free energy landscapes computed in high dimensions, reflecting the trend seen with the consistency of Target XXXII. Despite the number of conformers continuing to fluctuate as the largest data set size is reached, the average positions of equivalent conformers are fairly similar, as demonstrated by Figure 6b. For the landscapes in vacuum and chloroform, equivalent conformers are found on average less than 1.2 Euclidean radians from each other in a 7-dimensional space. This number is slightly less consistent in DMSO, with some Δ*d̅* values being as high as 1.8 Euclidean radians, even toward the final data set size. Finally, considering the difference in free energies between equivalent conformers, demonstrated in Figure 6c, it can be seen that in all three environments, conformers deemed equivalent are within, on average, 5 kJ mol^–1^ of each other. This is not as good an agreement as observed in the lower-dimensional cases; however, it will be sufficient when comparing the free energies of conformers that differ by more than this amount.

**Figure 5:**
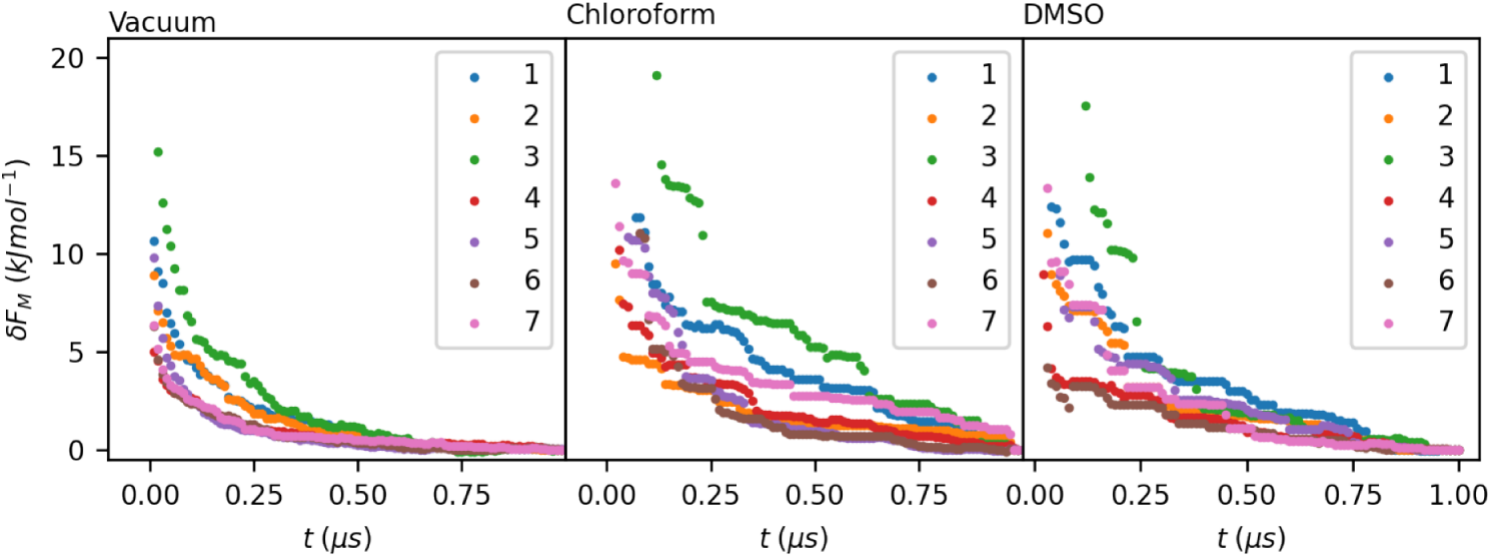
Evolution of δ*F*(*t*) on the marginal free energies of each torsion in bicalutamide, in vacuum, chloroform, and DMSO.

**Figure 6:**
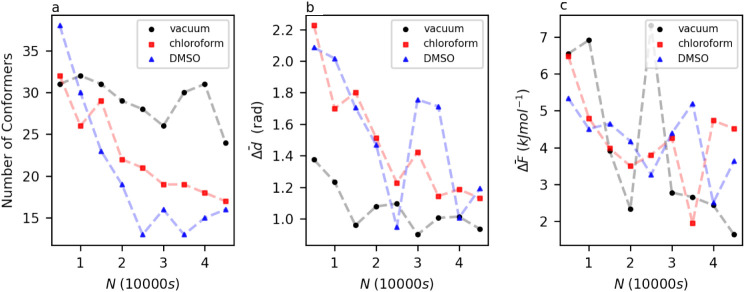
a: Number of clusters identified by clustering on data sets of size *N* for bicalutamide, in vacuum, chloroform, and DMSO. b: Evolution of Δ*d̅* with *N* for bicalutamide, in vacuum, chloroform, and DMSO. c: Evolution of Δ*F̅* with *N* for bicalutamide, in vacuum, chloroform, and DMSO.

The per-point free energy landscapes generated through the gridless analysis are projected into two dimensions using Sketch-map. To enable comparison across different environments, the Sketch-map projections generated for the solvated cases use the same *a*, *b*, and σ parameters and landmark points as determined from the postprocessing of the simulation in vacuum. This ensures that the resulting two-dimensional projection is equivalent across the different environments. These projections in vacuum, chloroform, and DMSO are shown in Figure 7a,b,c, respectively. For each of the projections, an enlarged image featuring the locations of the free energy minima marked with a numerical label is available in Figures S11–S13. These labels correspond to the conformer indices in the left-hand columns of Tables S1–S3 for bicalutamide in vacuum, chloroform, and DMSO, respectively. Some of the structure present in the vacuum projection seems to be preserved in the chloroform projection, with the left–right gulf having been narrowed slightly and conformers being distributed more diffusely. The DMSO projection, however, appears significantly different, with a new network of interconnected conformers being shown.

**Figure 7:**
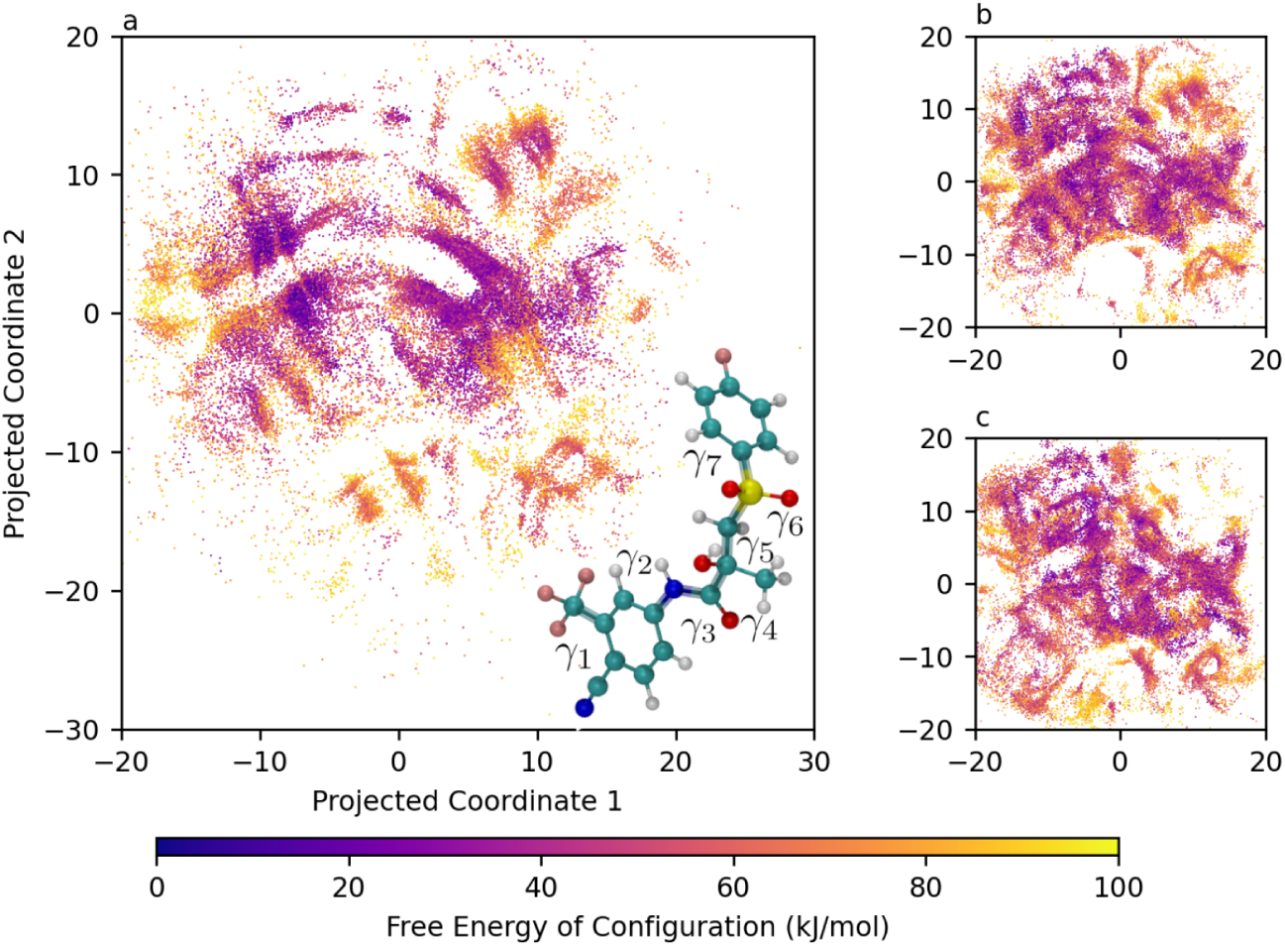
2D Sketch-Map projection of bicalutamide’s 7D conformational free energy landscape in vacuum (a), chloroform (b), and DMSO (c), with molecular structure of bicalutamide inset. Distances between configurations are preserved over small separations but the axes themselves have no physical meaning.

Figure 9 compares the conformational free energy minima in each solvent environment with the experimentally determined crystal structures. Figure 9a shows the most stable conformer in DMSO, which has an open conformation, as observed experimentally by Sobornova et al.^39^ This conformation is distinct but similar to the conformation observed in form I of bicalutamide, illustrated in Figure 9c. The most stable conformer in chloroform is shown in Figure 9b and demonstrates a closed conformation, again matching the experimental observation of Sobornova et al.^39^ Additionally, the conformation adopted in chloroform corresponds closely with the conformation adopted in crystal form II, shown in Figure 9d.

With confirmation that the most stable conformers in each environment align with experimental observations, a more extensive analysis of the relationships between distinct conformers and solvent environments can be carried out. Figure 8 shows a double matrix comparing all bicalutamide conformers in chloroform to all bicalutamide conformers in DMSO. Each element’s number corresponds to the difference in free energy between the two conformers (relative to the most stable conformers in their environment). The color gradient indicates the similarity of the structures, measured by their minimum RMSD separation^41,42^ considering all atom positions. In order to study these conformer sets more closely, we define the set of common conformers to be those conformer pairs that exhibit a minimum RMSD of less than 1.7 Å. There are 10 of these common conformers for bicalutamide in chloroform and DMSO, and they are shown in Figure 10. The overlapped conformers are shown in blue for chloroform and red for DMSO. It is interesting to note that the fully closed conformation observed as the most stable form in chloroform and in bicalutamide’s crystalline form II, shown in Figure 9b,d, is not present as a common conformer, meaning it does not arise at all in DMSO. The majority of the common conformers shown in Figure 10 seem to exhibit a semiopen “L”-shaped conformation, rather than the fully open and closed conformations seen as the most stable conformers in DMSO and chloroform in Figure 9a,b. The conformers in Figure 10 are ordered by Δ*F* where

$$\Delta F=F_{\mathrm{CLF}}-F_{\mathrm{DMSO}}$$

**Figure 8:**
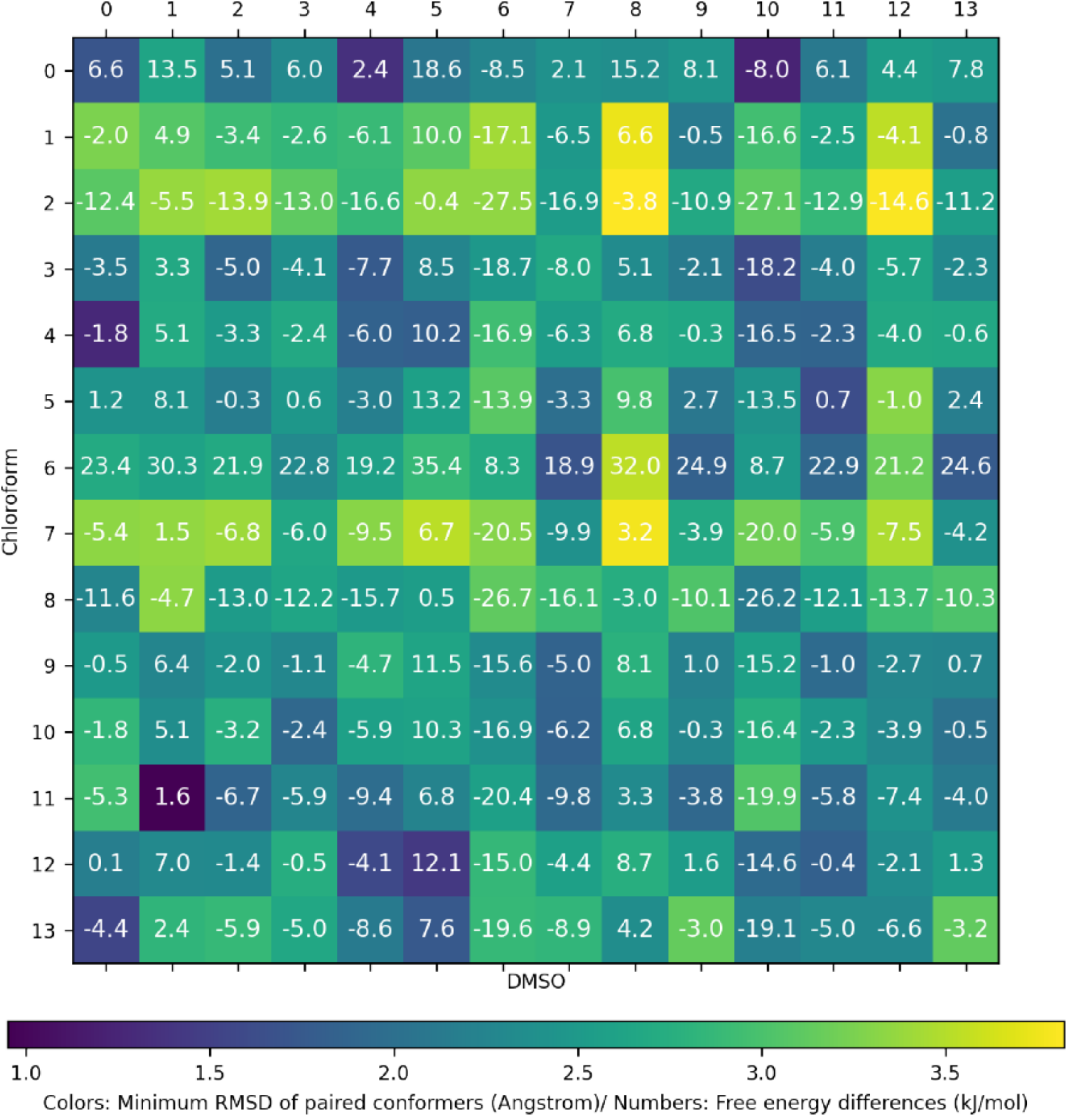
A dual matrix presenting a pairwise comparison of conformers of bicalutamide observed in chloroform (rows) and DMSO (columns). The color gradient indicates the minimum RMSD between atoms between the two conformers, while the number within each element indicates the stability of the conformer in chloroform relative to the conformer in DMSO.

**Figure 9:**
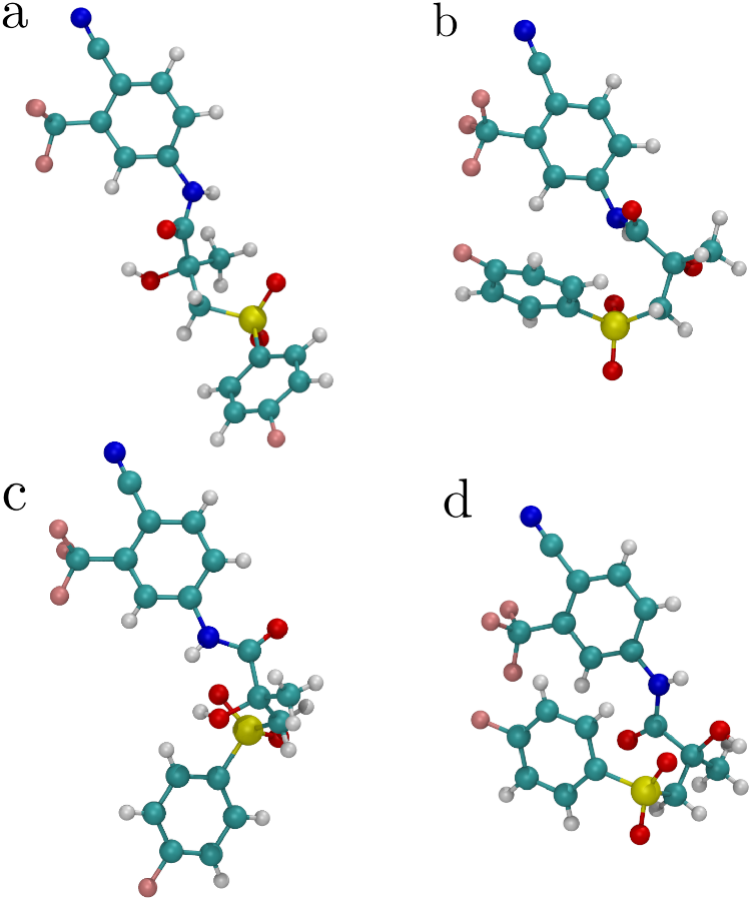
a: The lowest free energy conformer of bicalutamide in DMSO, index 5 Figure 7b. b: The lowest free energy conformer of bicalutamide in chloroform, index 2 in Figure 7c. c: The experimentally observed conformation of bicalutamide form I. d: The experimentally observed conformation of bicalutamide from II.

**Figure 10:**
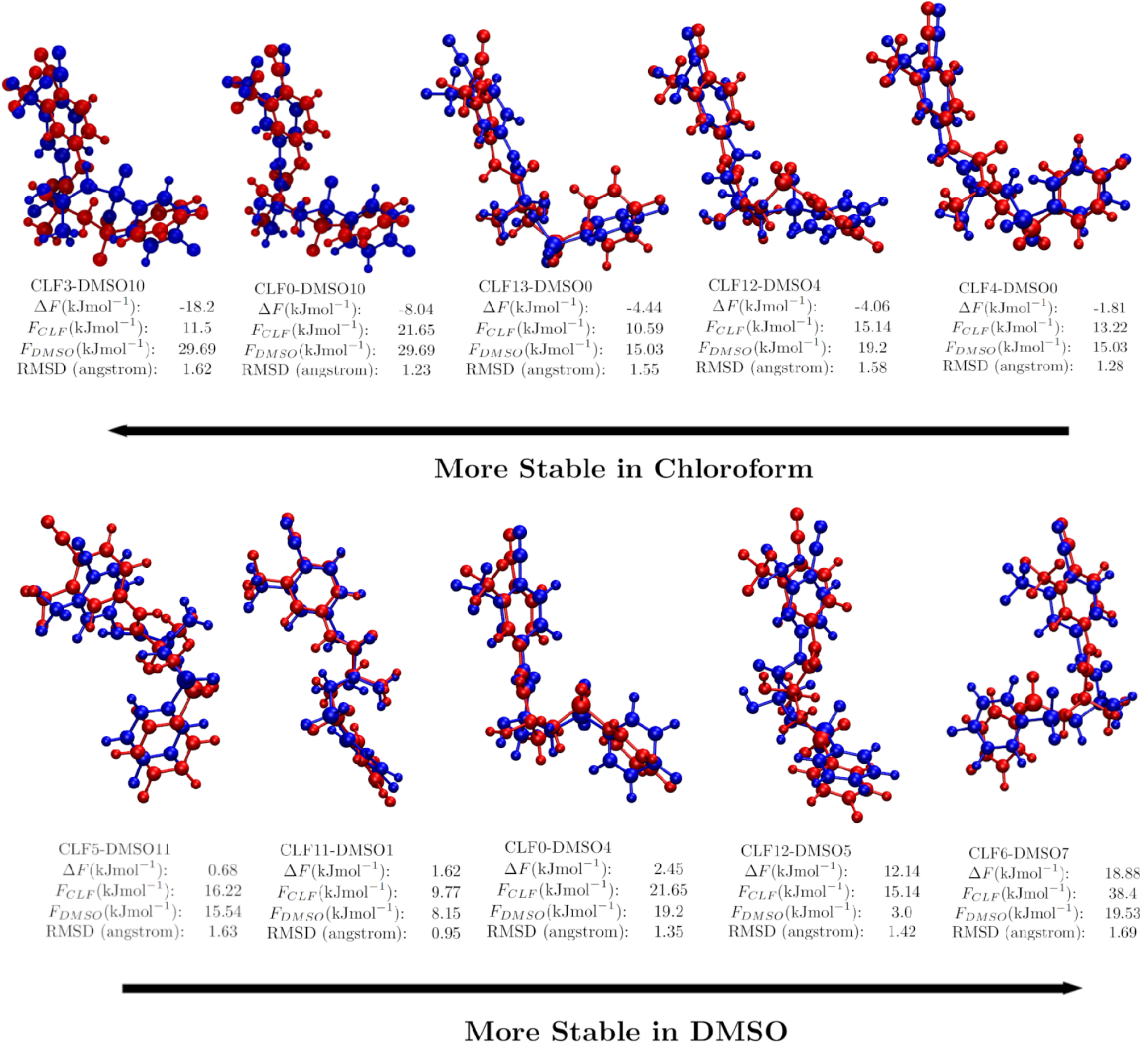
Ten common conformers of bicalutamide in chloroform and DMSO. For this system, conformers are deemed common to both solvents if their overlaid structures present a minimum RMSD deviation of less than 1.7 Å. Conformers in chloroform are indicated with the label CLF and have their molecular structures shown in blue. Conformers in DMSO are indicated with the label DMSO, and their molecular structures are shown in red. The free energy in both solvents, as well as the difference in free energy, is indicated for each conformer, and the conformers are ordered from those most stabilized in chloroform to those most stabilized in DMSO.

where *F*_CLF_ and *F*_DMSO_ are the free energies of the conformers in chloroform and DMSO, respectively. Note that, as before, these individual free energies are themselves relative to the lowest energy configuration within the free energy landscape.

Figure 10 thus seems to show that these “L”-shaped conformers tend to be stabilized in chloroform, the same environment that promotes the fully closed conformer, and that common conformers with a greater open character tend to be stabilized by DMSO.

It is also interesting to consider the common conformer labeled CLF5-DMSO11, corresponding to conformer 5 in the chloroform landscape and 11 in the DMSO landscape. This conformation closely resembles bicalutamide crystal form I, as shown in Figure 9c. This common conformer has a low Δ*F* of 0.68 kJ mol^–1^, indicating that it is equally stabilized by both solvents, but has an *F*_CLF_ of 16.22 kJ mol^–1^ and an *F*_DMSO_ of 15.54 kJ mol^–1^, making it far from the most stable conformer in either solvent. From this, it can be inferred that while the form I conformation is metastable in solution, it is not purely a solvent effect that is responsible for the conformational rearrangements leading to the observed conformational polymorph.

## Conclusions

We have introduced a gridless methodology for constructing high-dimensional conformational free energy landscapes from enhanced sampling simulations. By leveraging concurrent well-tempered metadynamics and Density Peaks Advanced (DPA) clustering, our approach enables the assignment of per-configuration free energies without resorting to dimensionality reduction or binning. This framework avoids the exponential cost of grid-based FES construction, making it particularly well-suited for flexible, drug-like molecules.

We validated the method across a range of molecular systems, from the benchmark alanine dipeptide to realistic pharmaceutical compounds with up to 11 torsions. Crucially, we demonstrated its application in explicit solvent environments, capturing solvent-induced shifts in conformational preferences of bicalutamide in quantitative agreement with experimental observations. The consistent performance across increasing dimensionality, together with the ability to capture solvent effects, underscores the robustness and transferability of the proposed approach.

Naturally, these features come with practical trade-offs. Generating sufficiently ergodic sampling for fully solvated systems requires molecular dynamics simulations on the scale of tens to hundreds of nanoseconds, which constrains the accuracy of the potential energy functions that can be feasibly employed. Moreover, because our approach relies on explicit sampling rather than energy minimization, it is inherently less suited for large-scale screening. While minimization-based workflows can be applied to thousands of compounds^9–12^ our method is suited to rigorously investigating how explicitly represented environments affect APIs’ conformational landscapes.

Because the method operates directly on torsional coordinates, it is agnostic to the underlying simulation engine or force field. As such, we envision its application in conjunction with machine-learned potentials as a key future development to complement extensive sampling with DFT-level accuracy. These features provide a practical and extensible tool for exploring conformational thermodynamics in atomistic simulations of complex molecular systems. The code developed here is fully open source and available from https://github.com/ucecvan/Twister.

## Supplementary Material



## Notes

Supporting figures reporting sketchmap representations at different data set sizes, and tables summarizing free energy and position in dihedral angle space of bicalutamide conformers in various environments (PDF)

## #

Particology Ltd., Discovery Park House, Ramsgate Road, Sandwich CT13 9ND, United Kingdom
