# Supplementary material for: Sampling High-Dimensional Conformational Free Energy Landscapes of Active Pharmaceutical Ingredients
Source: J Chem Theory Comput. 2025 Dec 4;21(24):12466–80. doi: 10.1021/acs.jctc.5c01247 (PMC12746462; doi:10.1021/acs.jctc.5c01247)
Supplement: Supplementary file 1 [file ct5c01247_si_001.pdf]

Supplementary Materials for: Sampling High-Dimensional Conformational Free Energy Landscapes of Active Pharmaceutical Ingredients

Alexandre Ferreira,[1] Rui Guo,[2] Ivan Marziano,[2,3] and Matteo Salvalaglio*[1]

[1] *Thomas Young Centre and Department of Chemical Engineering, University College London, London, WC1E 7JE, United Kingdom.*

[2] *Pfizer Worldwide Research & Development, Sandwich CT13 9ND, United Kingdom.*

[3] *Present Address: Particology Ltd., Discovery Park House, Ramsgate Road, Sandwich CT13 9ND, United Kingdom.*

(*m.salvalaglio@ucl.ac.uk)

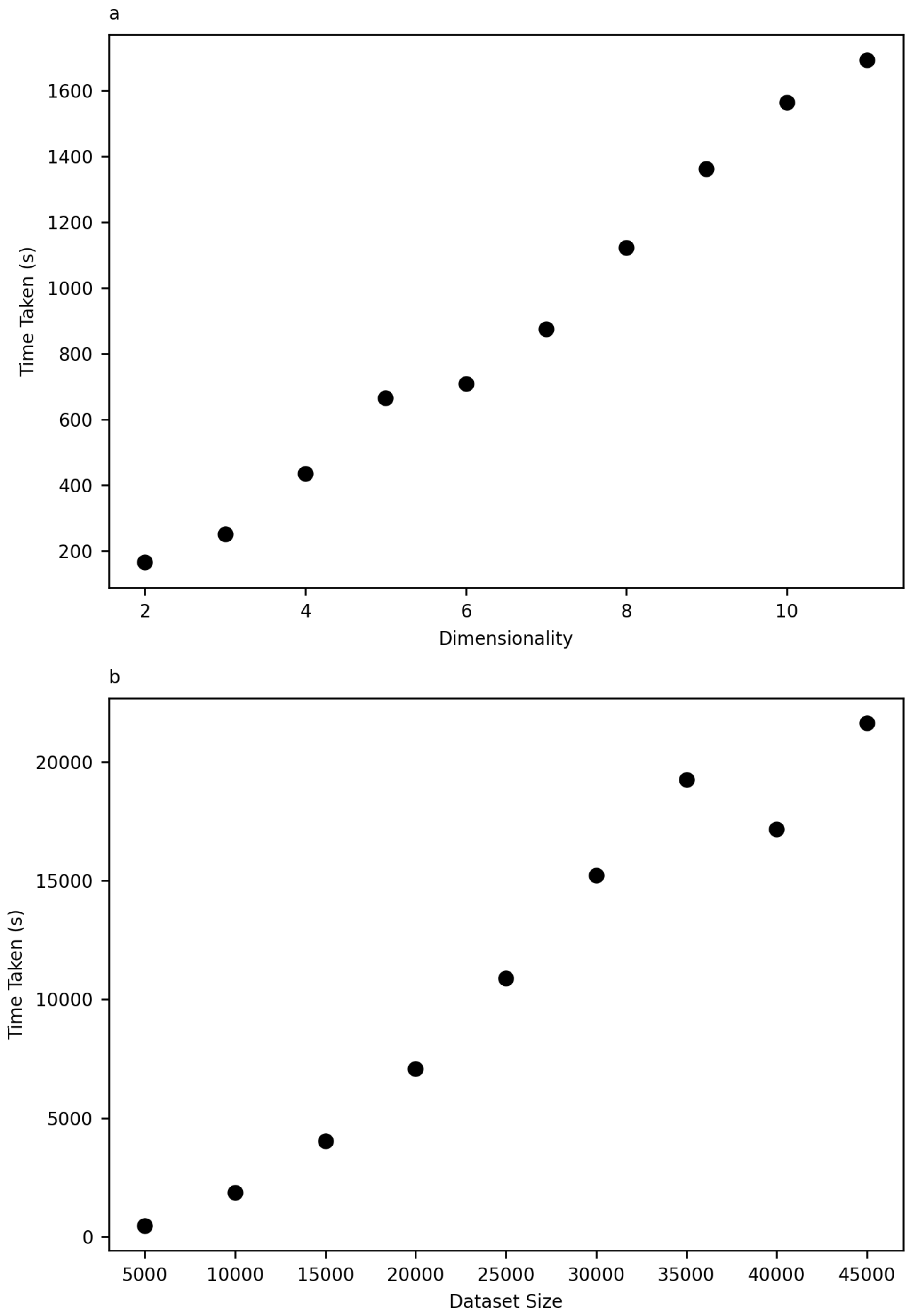

Figure S1: Computational cost of the analysis procedure as it increases with (a): the dimensionality of conformation space, on a dataset with a constant size of 10,000 molecular configurations, and (b): the size of 11-dimensional configurational datasets. Cost is shown as the time taken to carry out the analysis on a standard desktop workstation. The cost of the simulation used to generate the configurations is not shown. Configurations of Target XXXII are used in both cases. As expected, the cost increases linearly with dimensionality, and with the square of dataset size.

TABLE S1: Labels, free energies and CV-space coordinates of bicalutamide's conformers in vacuum. The labeling convention is consistent with that of Figure S11

Conformer	Free Energy [kJ/mol]	γ_1	γ_2	γ_3	γ_4	γ_5	γ_6	γ_7
0	8.16	-1.9	-3.06	-3.13	1.13	-1.38	2.96	1.45
1	15.83	-2.0	2.54	-3.06	1.5	-0.64	1.3	-2.36
2	4.09	2.37	-2.47	3.06	-2.23	1.02	-1.11	2.03
3	0.0	-0.02	-2.42	-3.11	-2.16	1.22	-1.23	-1.17
4	19.0	0.04	-2.25	2.91	-2.18	-3.03	-2.82	-1.72
5	0.52	2.11	-2.41	2.79	-2.23	1.23	-1.22	-1.07
6	1.17	-1.84	-2.41	2.86	-2.25	1.23	-1.15	-1.03
7	1.28	0.04	-2.65	2.85	-2.25	1.23	-1.05	1.95
8	21.5	-2.07	-2.64	2.87	-2.3	-3.01	-2.83	1.39
9	14.63	0.14	2.21	-2.92	1.14	-1.58	2.88	1.25
10	23.18	1.83	2.94	3.07	-2.3	-3.05	-2.75	1.39
11	2.23	-2.16	-2.63	3.09	-2.29	1.21	-1.09	2.05
12	12.55	2.16	2.49	-3.03	1.4	-1.08	-2.47	-1.42
13	14.18	0.15	2.58	-2.91	1.72	-0.8	1.53	-2.09
14	11.29	2.12	2.66	-3.02	1.15	-1.46	2.85	1.56
15	11.6	-0.21	2.36	-3.05	1.0	-1.51	2.86	-1.76
16	25.23	-0.15	-2.53	2.85	-2.09	-3.01	-2.51	1.46
17	7.03	1.81	-2.58	2.94	-2.02	1.01	-1.23	2.41
18	15.88	0.04	2.47	-3.13	0.8	0.7	0.78	-2.02
19	14.32	1.91	2.58	3.07	1.58	-0.82	1.4	-2.2
20	17.52	-0.01	2.65	-3.02	1.49	-0.55	1.11	0.74
21	9.81	-2.13	2.68	3.09	1.29	-1.38	2.85	-1.14
22	28.67	0.14	1.68	-0.12	1.36	0.68	0.95	-1.9
23	3.07	-2.08	-2.43	2.77	-2.08	0.99	-1.36	2.08
24	15.28	1.94	2.52	3.11	1.73	-0.82	1.45	0.92

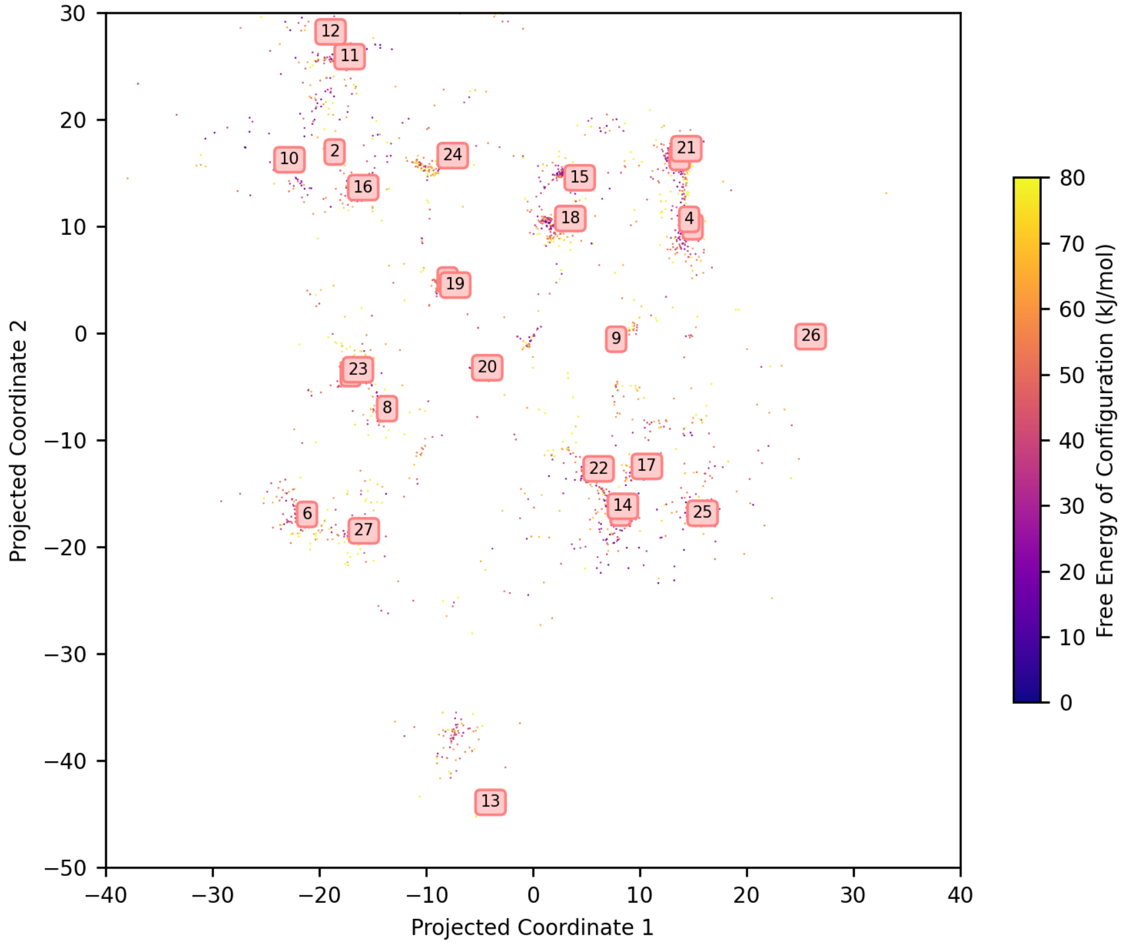

Figure S2: 2D Sketchmap projection of the 11D per-point FES of Target XXXII generated from a dataset of 5000 configurations. Shown here alongside other projections of Target XXXII's FES generated with smaller datasets, to illustrate the evolution of features of the FES as the size of the dataset increases.

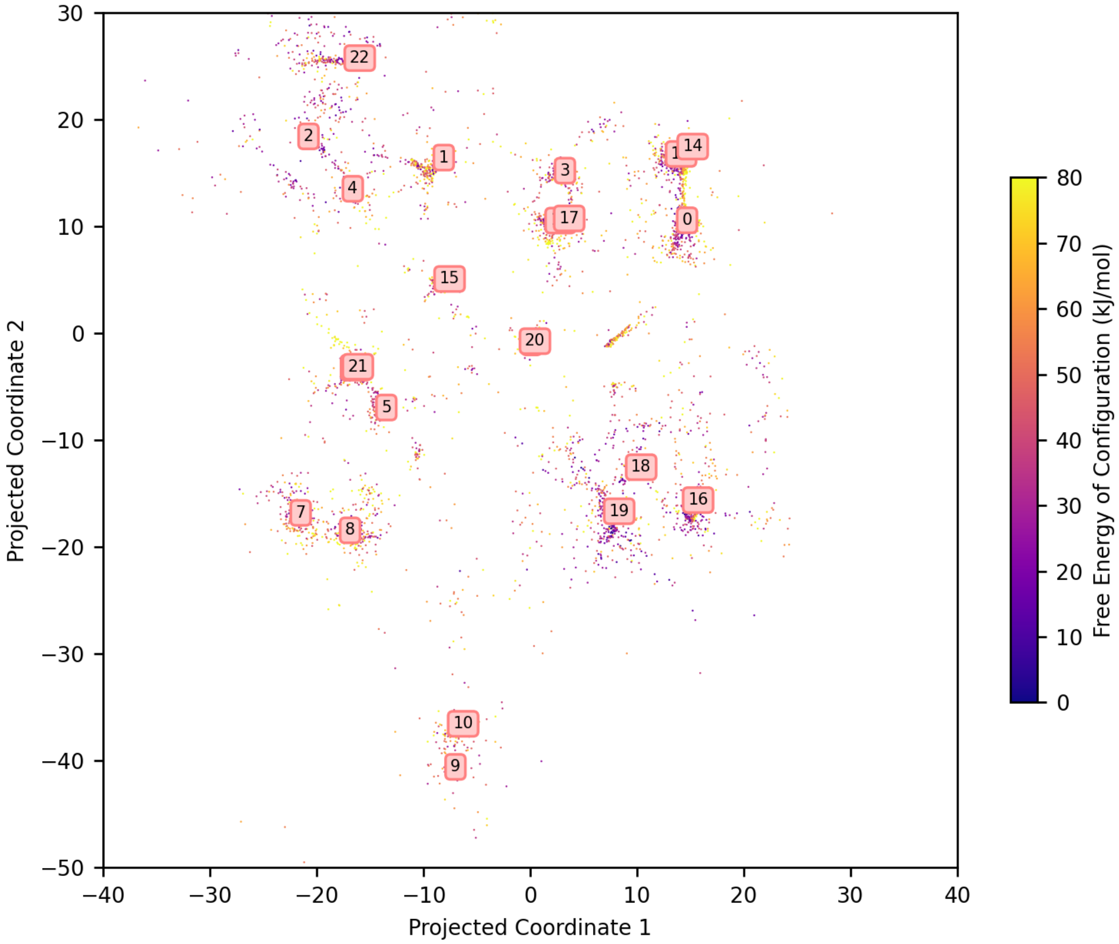

Figure S3: 2D Sketchmap projection of the 11D per-point FES of Target XXXII generated from a dataset of 10000 configurations. Shown here alongside other projections of Target XXXII's FES generated with smaller datasets, to illustrate the evolution of features of the FES as the size of the dataset increases.

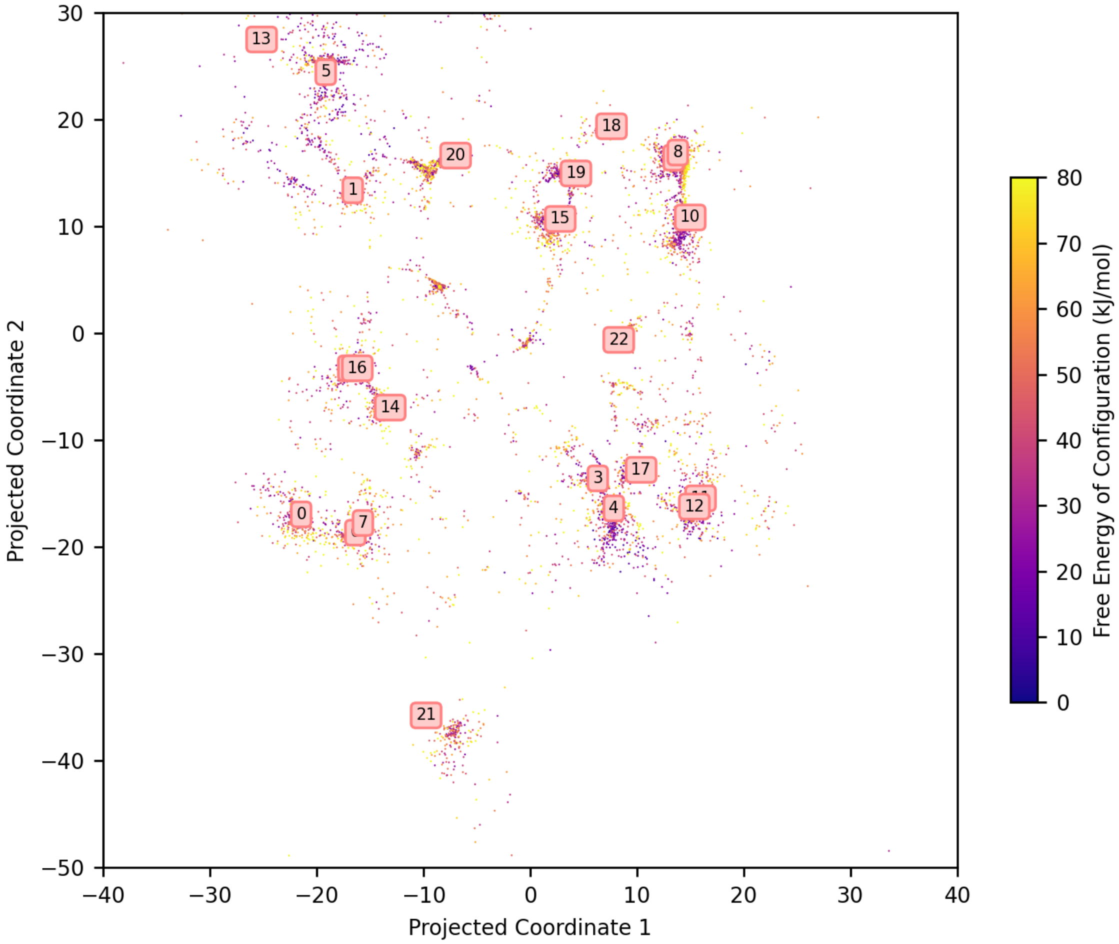

Figure S4: 2D Sketchmap projection of the 11D per-point FES of Target XXXII generated from a dataset of 15000 configurations. Shown here alongside other projections of Target XXXII's FES generated with smaller datasets, to illustrate the evolution of features of the FES as the size of the dataset increases.

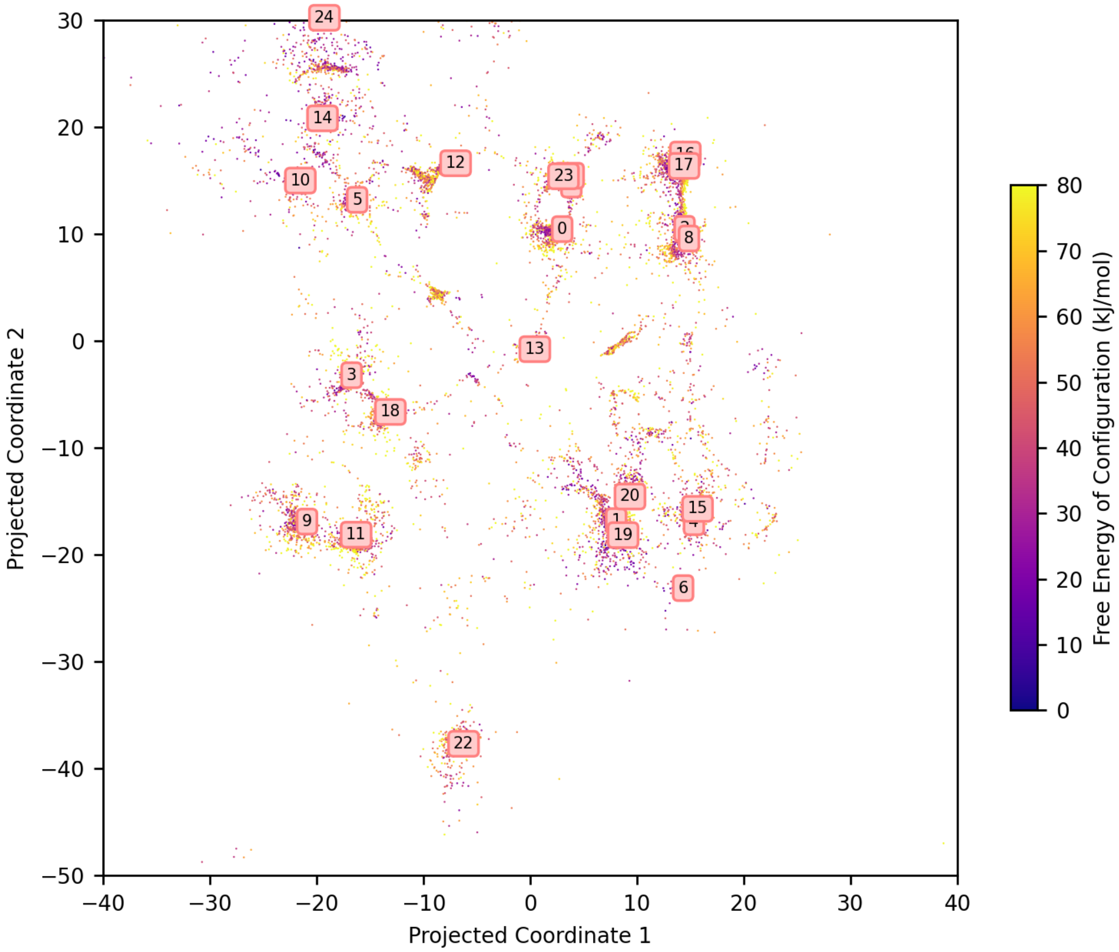

Figure S5: 2D Sketchmap projection of the 11D per-point FES of Target XXXII generated from a dataset of 20000 configurations. Shown here alongside other projections of Target XXXII's FES generated with smaller datasets, to illustrate the evolution of features of the FES as the size of the dataset increases.

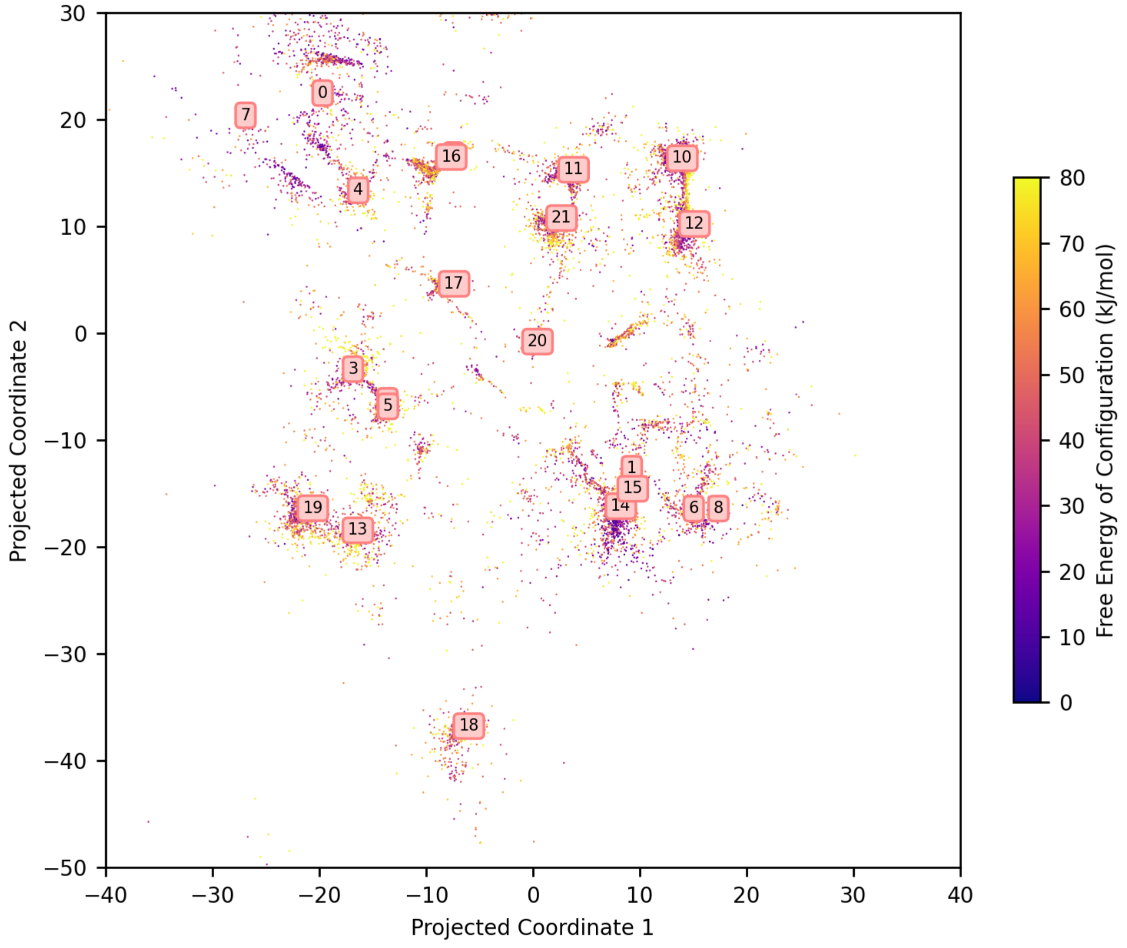

Figure S6: 2D Sketchmap projection of the 11D per-point FES of Target XXXII generated from a dataset of 25000 configurations. Shown here alongside other projections of Target XXXII's FES generated with smaller datasets, to illustrate the evolution of features of the FES as the size of the dataset increases.

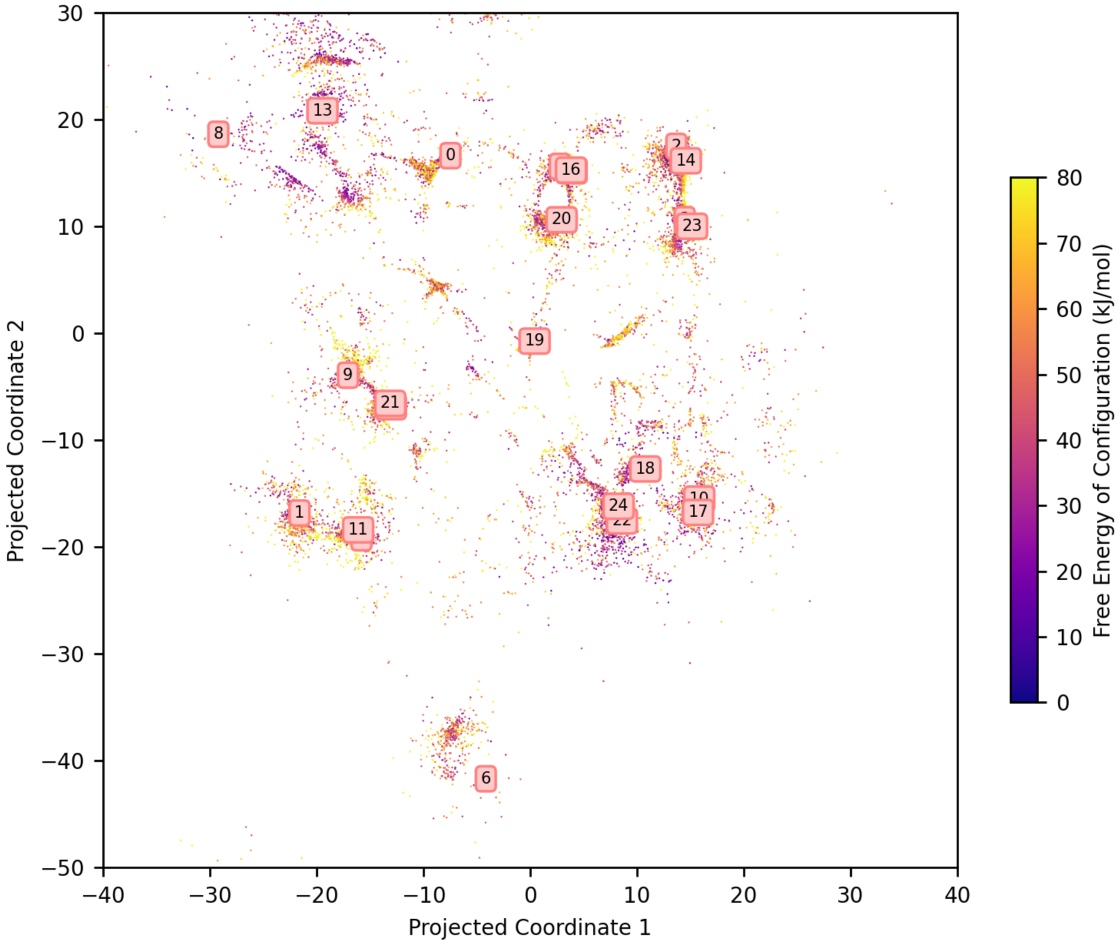

Figure S7: 2D Sketchmap projection of the 11D per-point FES of Target XXXII generated from a dataset of 30000 configurations. Shown here alongside other projections of Target XXXII's FES generated with smaller datasets, to illustrate the evolution of features of the FES as the size of the dataset increases.

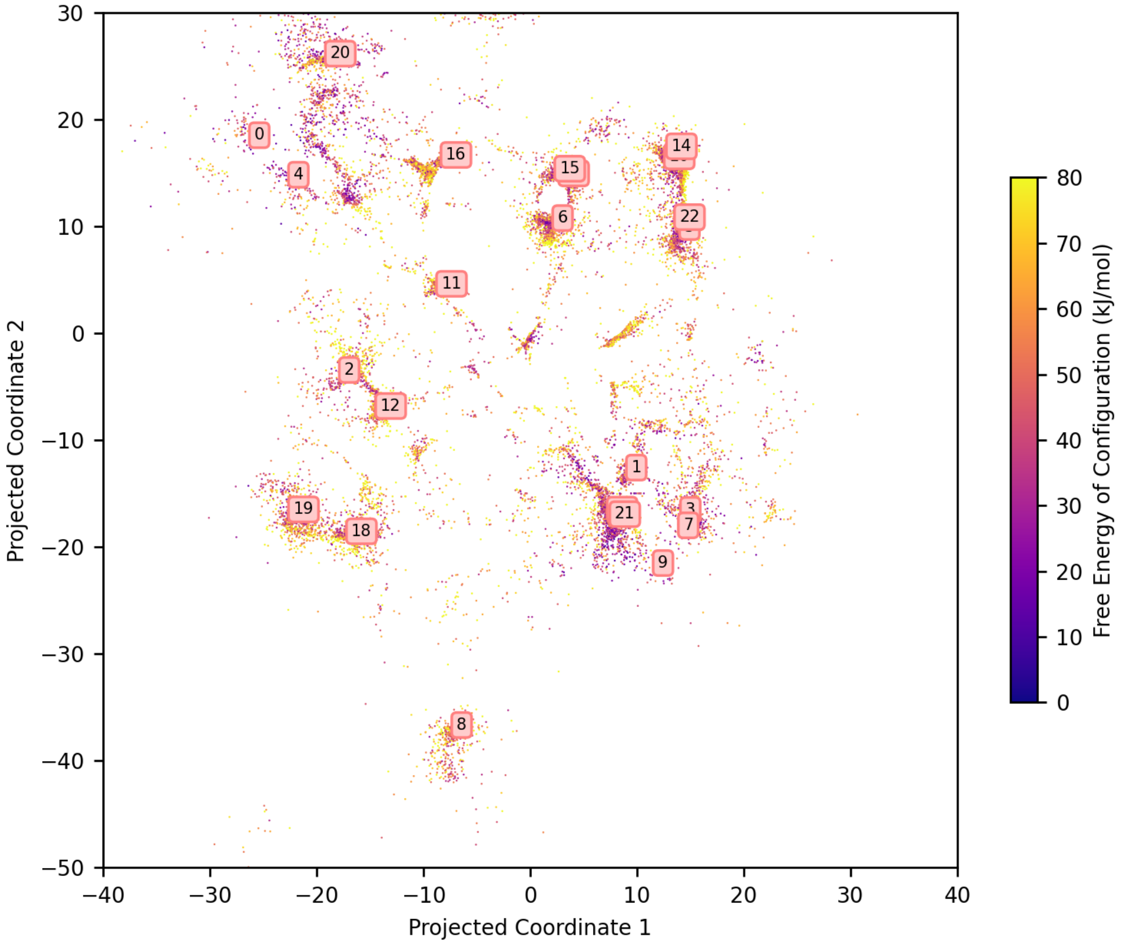

Figure S8: 2D Sketchmap projection of the 11D per-point FES of Target XXXII generated from a dataset of 35000 configurations. Shown here alongside other projections of Target XXXII's FES generated with smaller datasets, to illustrate the evolution of features of the FES as the size of the dataset increases.

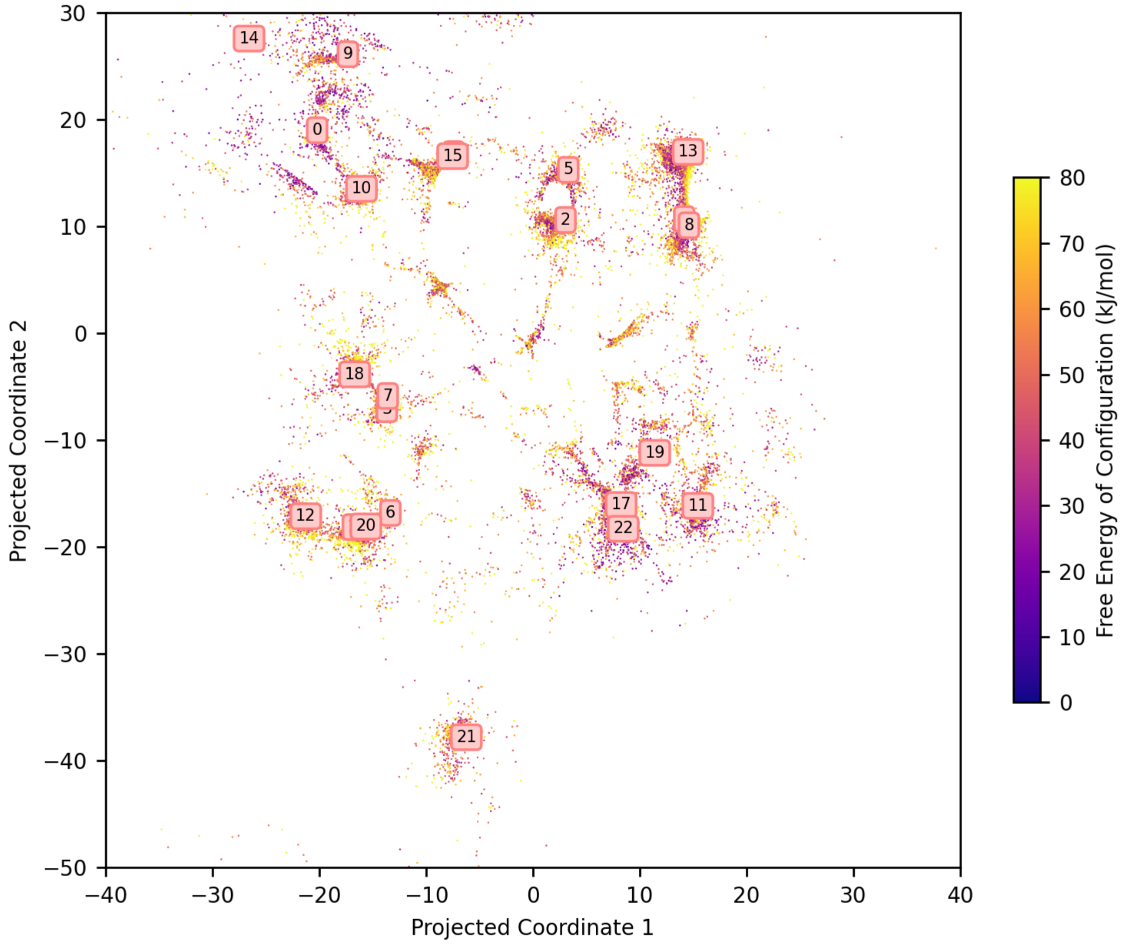

Figure S9: 2D Sketchmap projection of the 11D per-point FES of Target XXXII generated from a dataset of 45000 configurations. Shown here alongside other projections of Target XXXII's FES generated with smaller datasets, to illustrate the evolution of features of the FES as the size of the dataset increases.

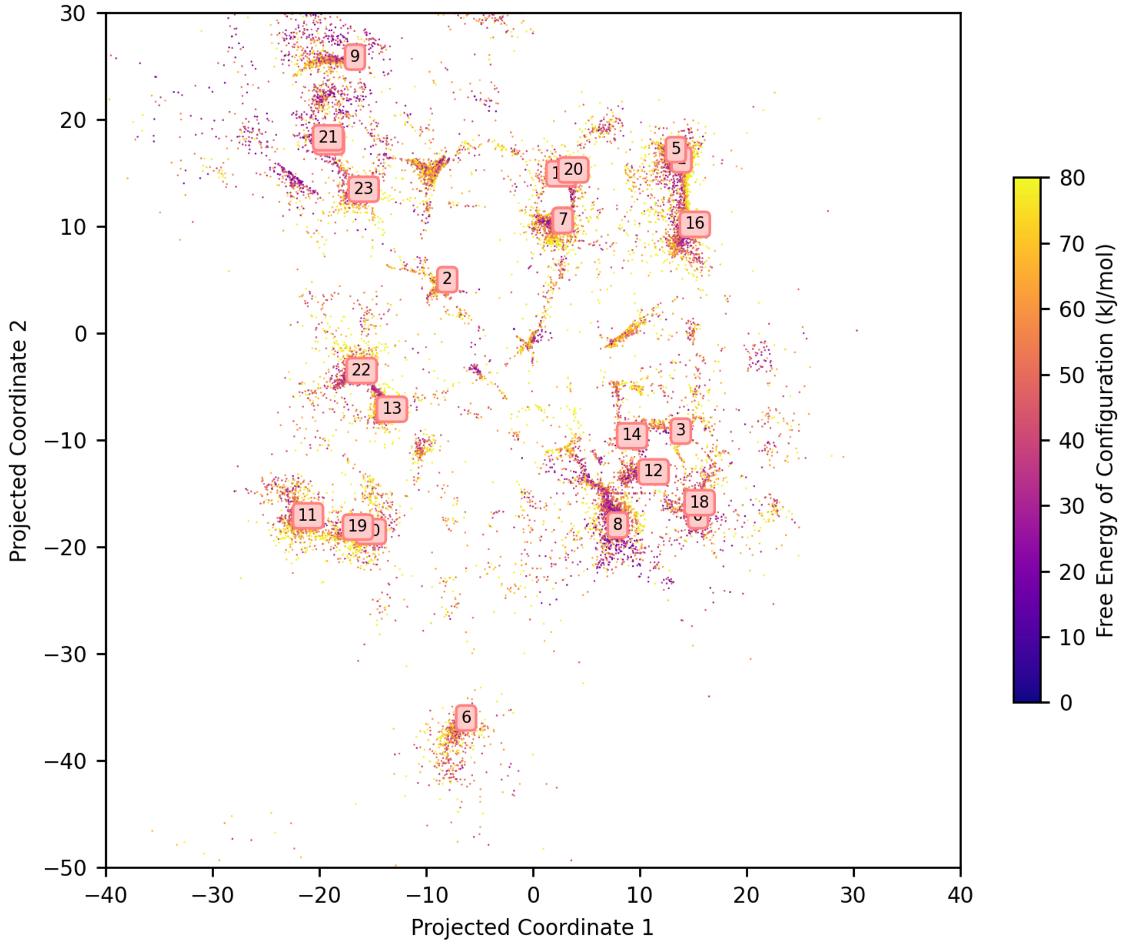

Figure S10: 2D Sketchmap projection of the 11D per-point FES of Target XXXII generated from a dataset of 45000 configurations. Shown here alongside other projections of Target XXXII's FES generated with smaller datasets, to illustrate the evolution of features of the FES as the size of the dataset increases.

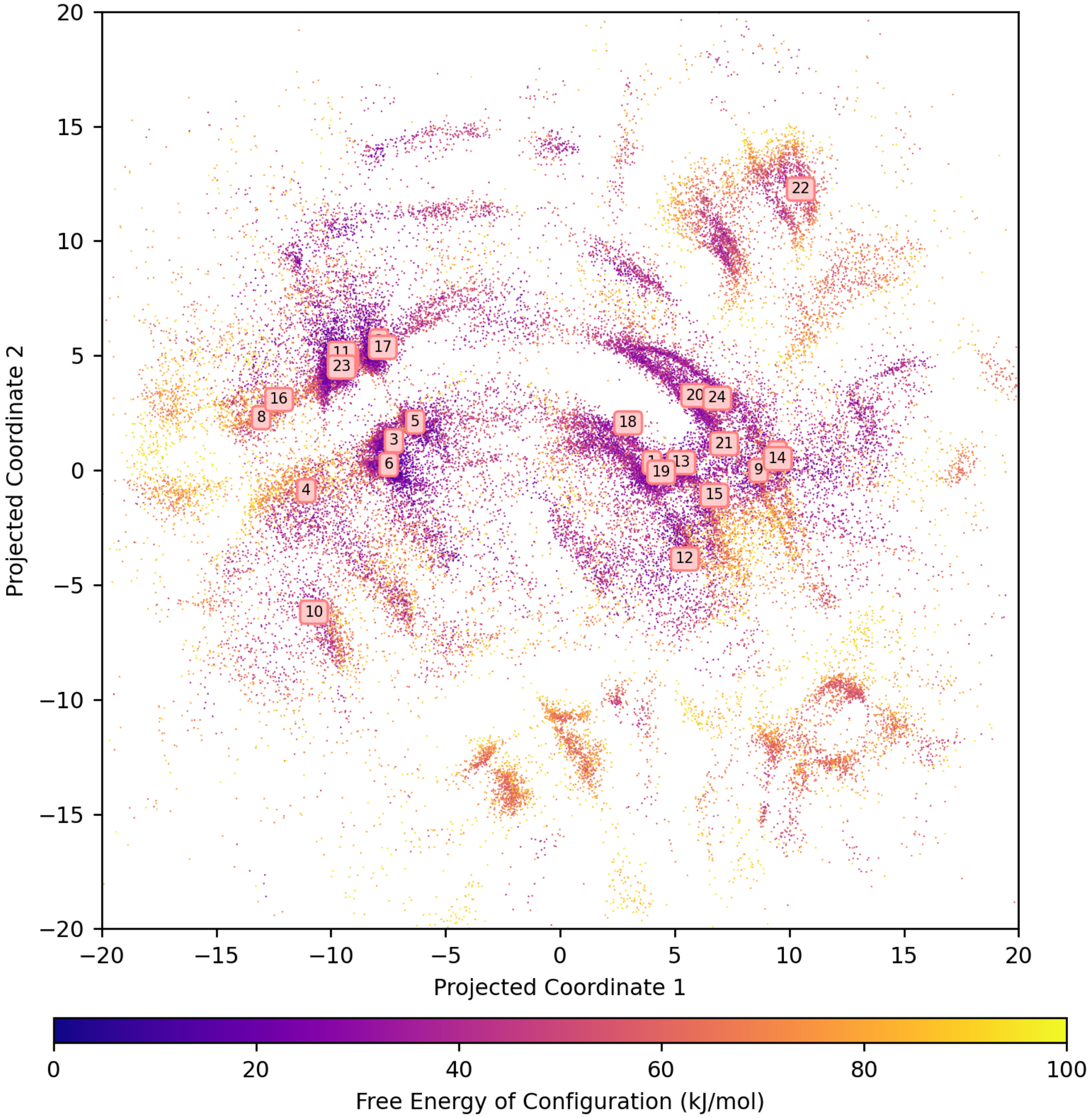

Figure S11: 2D Sketch-map projection of bicalutamide's 3D conformational free energy landscape in vacuum, with molecular structure of bicalutamide inset. Distances between configurations are preserved over small separations but the axes themselves have no physical meaning.

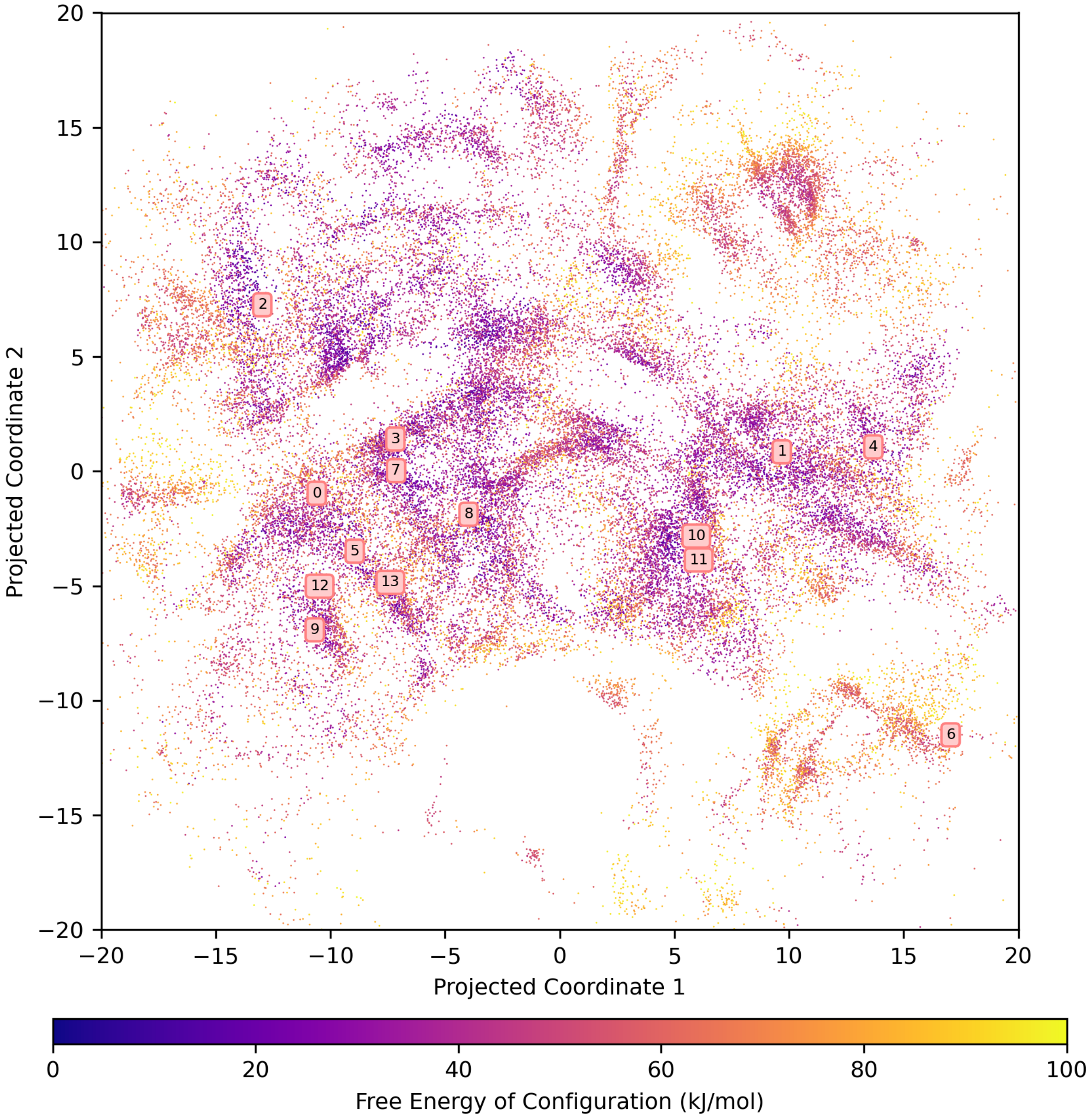

Figure S12: 2D Sketch-map projection of bicalutamide's 3D conformational free energy landscape in chloroform. Distances between configurations are preserved over small separations but the axes themselves have no physical meaning.

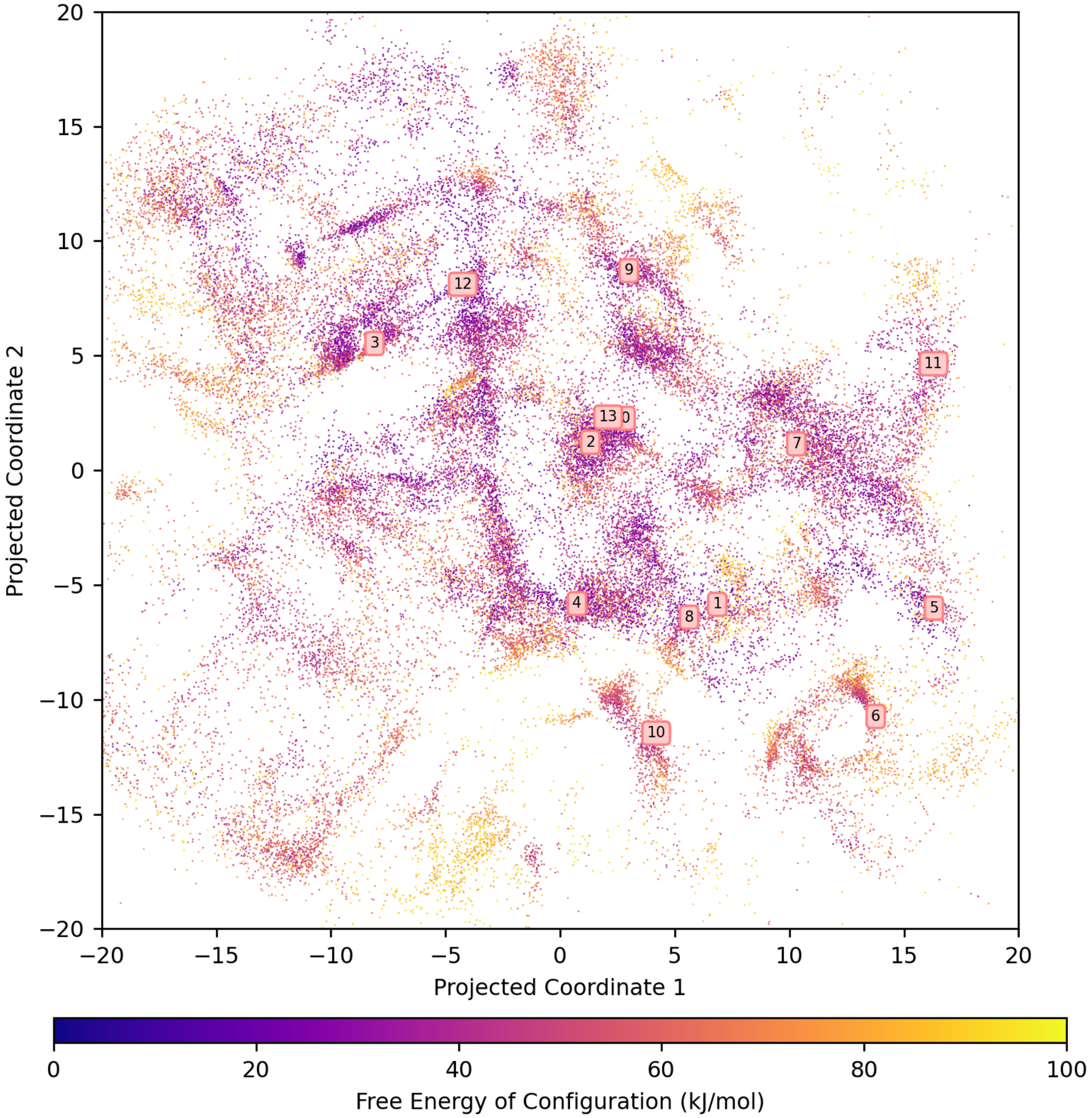

Figure S13: 2D Sketch-map projection of bicalutamide's 3D conformational free energy landscape in DMSO. Distances between configurations are preserved over small separations but the axes themselves have no physical meaning.

TABLE S2: Labels, free energies and CV-space coordinates of bicalutamide's conformers in chloroform. The labeling convention is consistent with that of Figure S12

Conformer	Free Energy [kJ/mol]	γ_1	γ_2	γ_3	γ_4	γ_5	γ_6	γ_7
0	21.65	-0.46	-2.17	2.77	-2.16	3.09	-3.02	-1.24
1	13.05	-2.12	2.77	2.93	1.45	-0.79	-3.03	1.91
2	2.6	2.39	2.82	2.93	-2.5	1.11	2.95	1.56
3	11.5	0.11	-2.66	2.83	-2.51	1.36	-0.75	-1.33
4	13.22	-2.29	-1.15	-3.12	1.23	-0.57	-3.08	1.8
5	16.22	-1.98	2.64	3.11	-1.97	-2.9	-2.71	-1.96
6	38.4	2.21	-1.34	-0.22	-1.1	-0.8	-0.92	1.76
7	9.66	-1.86	-2.42	-3.0	-2.27	1.24	-0.98	-1.07
8	3.47	-2.21	-2.75	-3.1	-2.7	0.98	2.53	-1.54
9	14.52	1.86	2.69	3.09	-1.96	-3.11	-2.9	1.72
10	13.28	0.26	-2.61	3.05	1.07	-1.64	2.88	-1.73
11	9.77	-2.23	2.54	3.03	1.52	-0.77	-2.79	-1.23
12	15.14	-2.23	-2.82	-2.99	-2.21	-3.1	-2.97	1.48
13	10.59	2.19	2.57	-3.01	-2.4	-2.97	-2.82	-1.59

TABLE S3: Labels, free energies and CV-space coordinates of bicalutamide's conformers in DMSO. The labeling convention is consistent with that of Figure S13

Conformer	Free Energy [kJ/mol]	γ_1	γ_2	γ_3	γ_4	γ_5	γ_6	γ_7
0	15.03	0.02	2.55	-3.07	0.88	0.89	0.9	-1.75
1	8.15	-0.24	-2.66	-2.9	-1.09	-0.82	-3.02	-1.64
2	16.5	2.21	-2.34	-3.01	1.0	0.83	0.88	-1.85
3	15.63	2.04	-2.59	2.93	-2.46	1.29	-0.92	2.09
4	19.2	0.1	-2.53	-3.08	2.77	-1.14	-1.69	-1.29
5	3.0	2.14	-0.61	3.04	-1.04	-1.02	2.95	1.14
6	30.15	-0.65	-1.47	0.17	-1.44	-0.9	3.08	1.45
7	19.53	-0.01	2.65	2.98	3.08	-1.38	2.57	1.67
8	6.43	-2.25	-2.44	3.13	-0.99	-0.9	-2.95	-1.76
9	13.55	-2.09	-0.93	-3.08	1.16	0.7	0.88	1.34
10	29.69	2.09	1.84	0.27	-1.29	-0.82	3.11	-1.61
11	15.54	0.03	-0.81	-3.07	2.47	-1.16	2.58	-1.27
12	17.2	1.86	-2.77	-3.13	3.11	-1.36	-1.6	2.21
13	13.81	-0.12	-2.58	-3.1	0.98	0.84	0.91	-1.63
